# Burden of malignant melanoma among the elderly population in East Asia from 1990 to 2023 and machine learning projection to 2040

**DOI:** 10.1371/journal.pone.0357767

**Published:** 2026-09-11

**Authors:** Qian Liu, Jianfeng Ma, Hao Yang, Na Liu, Chen Liu, Yiming Liu, Sheng Li, Yongfeng Wang

**Affiliations:** 1 Gansu Provincial Hospital & The Third Hospital of Lanzhou University, Lanzhou, Gansu, China; 2 School of Public Health, Lanzhou University, Lanzhou, Gansu, China; 3 Key Laboratory of Molecular Diagnostics and Precision Medicine for Surgical Oncology in Gansu Province, Gansu Provincial Hospital, Lanzhou, Gansu, China; 4 NHC Key Laboratory of Diagnosis and Therapy of Gastrointestinal Tumor, Gansu Provincial Hospital, Lanzhou, Gansu, China; 5 The First Clinical Medical College of Lanzhou University, Lanzhou, Gansu, China; 6 The Second Hospital & Clinical Medical School, Lanzhou University, Lanzhou, Gansu, China; National Center for Chronic and Noncommunicable Disease Control and Prevention, Chinese Center for Disease Control and Prevention, CHINA

## Abstract

**Background:**

Malignant melanoma (MM) is an emerging public health concern in East Asia’s rapidly aging population. This study assessed the burden of MM among older adults aged ≥60 years from 1990 to 2023 and projected age-standardized rates to 2040.

**Methods:**

Using GBD 2023 data, temporal trends were evaluated using Joinpoint regression and age–period–cohort analysis, together with age-, sex-, and location-specific comparisons. Future trends were projected using a multi-model forecasting framework incorporating ARIMA-derived temporal features. Seven candidate models were compared for each location–indicator combination. Historical data were chronologically divided into an 80% training set (1990–2016) and a 20% independent testing set (2017–2023), with model selection performed using five-fold rolling time-series cross-validation within the training set. Prediction intervals were generated using residual bootstrap.

**Results:**

From 1990 to 2023, MM-related deaths among older adults in East Asia increased from 3,417.59 to 7,476.55, while DALYs increased from 116,499.14 to 196,356.14. Joinpoint analysis showed significant overall increases in ASIR and ASPR, whereas ASMR and ASDR exhibited only small, non-significant overall changes. This divergence is consistent with an important contribution from population aging and growth of the older population to the increasing absolute burden. Women generally had higher incidence and prevalence rates, whereas men had higher mortality and DALY rates. Projections to 2040 showed substantial variation across locations, with mortality and DALY rates expected to reach a plateau in several locations.

**Conclusion:**

The increasing MM burden and distinct sex and regional patterns require targeted early-detection strategies and equitable access to dermatological care. Future studies should use regional, subtype-specific cancer registries to evaluate ALM-specific burden and inform prevention strategies for East Asian populations.

## 1. Introduction

Malignant melanoma(MM), a highly aggressive and lethal skin cancer originating from melanocytes, poses a significant global health challenge [1]. Characterized by its propensity for early metastasis, melanoma exhibits a mortality rate four times higher than non-melanoma skin cancers [2]. While surgical resection can be curative for localized disease, many patients progress to advanced stages with distant metastases, leading to poor prognosis [3]. Immune checkpoint inhibitors represent a paradigm shift in cancer treatment; however, therapeutic responses remain variable due to factors like tumor heterogeneity, leaving a substantial unmet clinical need and imposing a heavy burden on patients and healthcare systems [4].

Globally, MM incidence has risen steadily over recent decades, with pronounced disparities across regions and ethnicities [5]. The highest rates are observed in predominantly Caucasian populations, such as Australasia and North America, where the disease is primarily driven by ultraviolet exposure.However, the epidemiological and clinical profile of melanoma in East Asia is strikingly different. In East Asian populations, the predominant subtype is Acral Lentiginous Melanoma (ALM), which typically presents on the palms, soles, and nail beds and is largely unrelated to sun exposure. This critical etiological difference means that Western-centric prevention strategies focusing heavily on UV protection are insufficient for this region, providing a strong rationale for why an East Asia-specific epidemiological study is urgently needed. Critically, the risk escalates significantly with age, and mortality rates continue to increase among the oldest patient groups [6]. This trend presents a particular concern for East Asia, which is experiencing one of the world’s most rapid demographic shifts toward an aging population. Similarly, nations in East Asia, including China, Japan, and South Korea, are undergoing an epidemiologic transition, with their cancer profiles increasingly resembling those of high-income countries [7]. The interplay of population growth, rapid aging, and a rising proportion of elderly individuals (≥60 years) creates substantial pressures on cancer prevention efforts, thereby exacerbating the anticipated regional burden of MM [8].

Despite this growing threat, comprehensive data on the epidemiology and disease burden of MM specifically within the elderly population in East Asia remain scarce. Existing studies, often based on earlier data, confirm substantial increases in MM burden over past decades, but they do not fully reflect the recent demographic and epidemiological changes. A comprehensive analysis of temporal trends in incidence, prevalence, mortality, and DALYs across East Asia, based on the most current data, represents a significant gap in the region-specific comparative literature. Furthermore, few studies have explored long-term projections of MM burden among older adults to anticipate future public health challenges [9].

To address these gaps and provide a comprehensive regional perspective, this study used the most recent GBD 2023 dataset. We aimed to: (1) quantify the burden of MM among older adults (≥60 years) in East Asia from 1990 to 2023; (2) characterize temporal trends and age-, sex-, and location-specific differences within East Asia; and (3) project ASIR, ASPR, ASMR, and ASDR to 2040 based on historical patterns. For long-term projections, we employed a multi-model forecasting framework that integrated ARIMA-derived temporal features with machine-learning algorithms, combining conventional time-series modeling with flexible nonlinear fitting. Model selection was performed using rolling time-series cross-validation, and prediction intervals were generated through residual bootstrap. Ultimately, these findings may inform targeted prevention and disease-management strategies in the context of rapid population aging in East Asia.

## 2. Methods

### 2.1 Data source

To map the time trends of MM, this research leverages the GBD 2023 data, assessing the disease burden in East Asia’s elderly from 1990 to 2023 and projecting its trajectory through 2040 [10]. The GBD database integrates a wide range of data sources, including census records, civil registrations, vital statistics systems, cancer registries, hospital information systems, health surveys, and epidemiological reports from 204 countries and territories. This extensive dataset allows for a systematic and standardized estimation of disease burden across regions and time periods.

In this study, malignant melanoma of the skin was defined according to the GBD 2023 cause classification, corresponding to ICD-10 category C43 (C43.0–C43.9). No primary data were collected, and the authors did not independently identify or adjudicate individual cases [11]. We extracted data on the incidence, prevalence, mortality, and DALYs associated with MM from the publicly accessible GBD Results Tool (https://ghdx.healthdata.org/gbd-results-tool). The study was exempted from ethical review because it relied exclusively on anonymized, open-access data [12].

### 2.2 Basic study variables

The study utilized GBD 2023 data on MM incidence, prevalence, mortality, and DALYs for East Asian countries and the regional average (1990–2023). DALYs, which combine years of life lost (YLLs) and years lived with disability (YLDs), were derived for the analysis. Age-standardized incidence, prevalence, mortality, and DALY rates (ASIR, ASPR, ASMR, and ASDR) were extracted directly from the GBD 2023 Results Tool. These estimates were standardized by GBD using the GBD standard population and are expressed per 100,000 population [13].

The analysis focused on four core metrics: the age-standardized incidence rate (ASIR), capturing new MM cases; the age-standardized prevalence rate (ASPR), representing the total disease burden; the age-standardized mortality rate (ASMR), reflecting MM-related fatality risk; and the age-standardized disability-adjusted life years rate (ASDR), quantifying comprehensive health loss from both premature death and disability [14].

These standardized indicators enable cross-national and temporal comparisons by minimizing the influence of demographic differences among countries.

### 2.3 Study analysis

All statistical analyses and predictive modeling were performed using R (v4.4.1), Joinpoint Regression Program (v5.0), and Python (v3.12.7). P-values < 0.05 were considered statistically significant.

#### 2.3.1 Preliminary analysis.

To assess trends in the MM burden over time, we quantified changes in the age-standardized rates (ASIR, ASPR, ASMR, ASDR) by calculating the estimated annual percentage change (EAPC). This metric serves as a summary measure of the overall trend direction, where an EAPC value and its 95% CI both above zero signify a significant increase, while values below zero indicate a significant decrease [15].

We employed R packages ggplot2 and sf for spatial mapping of MM burden distribution and comprehensive visualization of geographic-temporal disparities across all indicators. Comparative analysis of age- and sex-stratified rates between countries revealed demographic variations, while all data manipulation and graphical representation were accomplished using dplyr and ggplot2.

#### 2.3.2 Joinpoint regression analysis.

Joinpoint regression analysis was used to identify inflection points in the time trends of MM indicators and to characterize segment-specific changes. A log-linear regression model was fitted to each segment to estimate the annual percentage change (APC), whereas the overall trend across the full study period was summarized using the average annual percentage change (AAPC) and its 95% confidence interval (CI) [16].

This model enables a detailed characterization of MM trends and helps determine periods of acceleration or deceleration in disease burden. Specifically, we applied Joinpoint regression to ASIR, ASPR, ASMR, and ASDR to quantify the rate and direction of change across East Asia. Model selection was based on Monte Carlo permutation tests, ensuring statistical robustness.Statistical significance was set at p < 0.05. Narrow 95% CIs distant from zero indicated stable and reliable estimates, whereas wider intervals suggested greater uncertainty.

#### 2.3.3 The age–period–cohort (APC) model.

Age–period–cohort (APC) analysis was performed using the estimable-functions framework implemented in the National Cancer Institute APC Web Tool [17]. Because birth cohort is exactly determined by calendar period and age (cohort = period − age), the unrestricted linear effects of age, period, and cohort cannot be estimated independently. Accordingly, individual regression coefficients were not interpreted as uniquely identifiable or causal effects. Instead, the analysis focused on identifiable APC functions, including longitudinal age curves, period rate ratios (RRs), and cohort RRs. The APC results were interpreted descriptively and were not used to establish independent causal effects of age, period, or birth cohort.

Using a standardized configuration of 5-year age groups (from 45–49 up to 95 + years) and six period intervals demarcated between 1990 and 2023, we conducted independent analyses of mortality and DALYs through the APC model. This design was crucial for attributing observed changes in MM burden to three distinct sources: the physiological process of aging, broader period effects capturing healthcare progress, or unique cohort effects reflecting generational risk factors. The model’s outputs thus provide a scientific rationale for formulating targeted screening and prevention strategies appropriate for East Asia’s aging demographic.

#### 2.3.4 Machine learning projection model.

To project the MM burden from 2024 to 2040, we developed a multi-model forecasting framework augmented with ARIMA-derived features. To strictly prevent data leakage, the continuous time-series data from 1990 to 2023 were chronologically divided into an 80% training set spanning 1990–2016 and a 20% independent testing set covering 2017–2023. We engineered comprehensive time-series features based solely on historical data, including first-, second-, and third-order lag features, 3-year rolling window statistics such as the mean and standard deviation, and both linear and squared non-linear time trend features. All input features were normalized using Z-score standardization via StandardScaler, with parameters fitted exclusively on the training set to prevent future data leakage.

To capture both linear autocorrelations and non‑linear patterns, the feature set was further augmented using an ARIMA‑based feature. Specifically, an ARIMA (1,1,1) model was fitted to the historical training data, and its out‑of‑sample predictions were generated through a 5‑fold rolling cross‑validation strictly within the training set; these predictions were then added as an additional input feature (ARIMA‑stacking feature) for all downstream models. This design allows the subsequent models to benefit from ARIMA’s strength in characterizing linear trends and short‑term dependencies.

Seven candidate models were trained on the augmented feature set: ARIMA, Bayesian Ridge Regression, Random Forest, eXtreme Gradient Boosting (XGBoost), AdaBoost, Decision Tree, and K‑Nearest Neighbors (KNN). Model selection and hyperparameter optimization were conducted strictly within the training set using a 5‑fold TimeSeriesSplit rolling cross‑validation to preserve chronological order. The optimal model for each country–indicator combination was selected primarily by minimizing the mean RMSE during cross‑validation. Bayesian Ridge Regression achieved the lowest mean RMSE across most country–indicator combinations during 5-fold rolling cross-validation and was therefore selected as the optimal model for the majority of final projections. Notably, Random Forest achieved the best predictive performance for the mortality and DALY projections in Japan and was selected as the optimal model for those specific indicators. By introducing Gaussian priors for regression coefficients, Bayesian Ridge effectively controls model complexity, preventing the overfitting common in standard linear regression when applied to strong linear time-series trends.

#### 2.3.5 Model validation and evaluation.

To ensure the robustness and reliability of our machine learning projections, we implemented a rigorous two-stage validation strategy. First, the dataset was strictly partitioned chronologically, allocating 80% of the historical data (1990–2016) to the training set and the remaining 20% (2017–2023) to a completely independent testing set. This chronological split completely prevents data leakage, which is critical in time-series forecasting.

During the model selection and hyperparameter tuning phase, we applied a 5-fold TimeSeriesSplit rolling cross-validation strictly within the training set. This approach evaluates the models using a historical window training and future window validation mechanism, effectively avoiding the chronological disruption and data leakage associated with standard random K-fold cross-validation. Finally, after the optimal model was identified and refitted on the entire training set, its true generalization capability was assessed exclusively on the unseen independent testing set (2017–2023).

To quantitatively evaluate the predictive performance of the candidate models, four internationally recognized regression metrics were calculated based on the independent testing set. These metrics included the Root Mean Square Error (RMSE), which measures the overall magnitude of prediction errors; the Mean Absolute Error (MAE), representing the average absolute difference between predicted and observed values; the Coefficient of Determination (R²), which assesses the proportion of variance in the dependent variable explained by the model; and the Mean Absolute Percentage Error (MAPE), which evaluates the relative forecasting error independent of the data scale. The final optimal model for each country-indicator combination was selected primarily based on minimizing the mean RMSE during the rolling cross-validation phase, with the mean absolute percentage error (MAPE) and the coefficient of determination (R²) computed alongside to provide a comprehensive assessment of each model’s predictive accuracy and generalizability.

For machine-learning models, 95% prediction intervals were generated using a residual bootstrap approach. The residuals from the 5-fold time-series cross-validation within the training set were pooled to form an empirical error distribution. In each of 1,000 bootstrap iterations, a noise term was randomly drawn from this distribution and added to the point prediction at each forecast step, with the feature set updated recursively. The 2.5th and 97.5th percentiles of the simulated forecast paths were taken as the lower and upper bounds. For ARIMA, asymptotic confidence intervals were directly obtained from the fitted model.

## 3. Results

### 3.1 Deaths and DALYs of MM among the elderly population across East Asian countries in 1990 and 2023

Between 1990 and 2023, the estimated number of deaths from MM among the elderly population in East Asia more than doubled, increasing from 3,417.59 (95% UI: 2,322.51–4,588.55) to 7,476.55 (95% UI: 5,468.33–10,570.26). China accounted for the largest number of deaths in both years, increasing from 2,834.82 to 5,846.17. Japan and the Republic of Korea also showed increases, while Taiwan Province of China recorded the largest relative increase, with the number of deaths increasing more than threefold. Mongolia and the Democratic People’s Republic of Korea had comparatively lower death counts in both years, although their estimated numbers were higher in 2023 than in 1990. At the regional level, the ASMR increased slightly from 2.05 per 100,000 population (95% UI: 1.39–2.89) in 1990 to 2.17 (95% UI: 1.59–2.99) in 2023. The corresponding EAPC was 50.79% (95% CI: −122.45% to 304.07%); because the confidence interval included 0, the estimated overall trend was not statistically significant. The numbers of deaths among both males and females more than doubled. Male ASMR point estimates were higher than the corresponding female estimates in both 1990 and 2023, reaching 2.31 and 2.01 per 100,000 population, respectively, in 2023 (Table 1).

**Table 1:** Death cases and ASMR per 100,000 population of MM among the elderly population across East Asian countries in 1990 and 2023.

Characteristics	1990		2023		1990-2023
	Deaths.Cases.No.(95% UI)	ASMR per 100,000 No.(95% UI)	Deaths.Cases.No.(95% UI)	ASMR per 100,000 No.(95% UI)	EAPC(%) in ASMR No.(95% CI)
Eastern Asia	3417.59 (2322.51,4588.55)	2.05 (1.39,2.89)	7476.55 (5468.33,10570.26)	2.17 (1.59,2.99)	50.79 (−122.45,304.07)
People’s Republic of China	2834.82 (1848.81,3860.36)	0.33 (0.21,0.45)	5846.17 (4208.65,8497.41)	0.27 (0.19,0.39)	10.19 (−8.83,33.33)
Japan	366.21 (316.46,422.31)	0.23 (0.2,0.26)	947.64 (760.91,1131.7)	0.25 (0.21,0.29)	0.26 (−23.72,41.72)
Republic of Korea	105.83 (73.8,162.57)	0.34 (0.24,0.54)	325.84 (228.31,477.94)	0.34 (0.24,0.5)	54.14 (2.72,131.98)
Democratic People’s Republic of Korea	47.28 (30.64,68.19)	0.31 (0.2,0.46)	151.1 (93.55,221.27)	0.47 (0.29,0.69)	−24.92 (−60.7,40.44)
Mongolia	5.06 (2.21,7.99)	0.49 (0.22,0.75)	8.68 (5.75,13.23)	0.37 (0.24,0.57)	28.7 (2.7,51.9)
Taiwan (Province of China)	58.39 (50.59,67.14)	0.37 (0.32,0.42)	197.11 (171.16,228.72)	0.47 (0.41,0.55)	−17.58 (−34.61,4.7)
**Gender**					
Male	1653.03 (906.16,2538.99)	2.17 (1.34,3.26)	3679.06 (2312.48,5717.02)	2.31 (1.54,3.46)	35.66 (−181.39,353.71)
Female	1764.56 (1047.17,2992.61)	1.95 (1.12,3.18)	3797.49 (2276.71,5953.41)	2.01 (1.26,3.15)	69.53 (−154.69,493.06)

During the same period, the estimated number of DALYs due to MM among the elderly population in East Asia increased substantially from 116,499.14 (95% UI: 78,155.93–155,712.94) to 196,356.14 (95% UI: 143,764.79–283,053.36). China contributed the largest number of DALYs in both years, followed by Japan and the Republic of Korea. All included locations recorded higher DALY counts in 2023 than in 1990, with the Democratic People’s Republic of Korea showing the largest relative increase, followed by Taiwan Province of China. The regional ASDR increased slightly from 59.04 per 100,000 population (95% UI: 39.80–82.01) in 1990 to 62.45 (95% UI: 46.30–86.69) in 2023. The corresponding EAPC was 47.81% (95% CI: −126.51% to 299.89%); because the confidence interval included 0, the estimated overall trend was not statistically significant. The number of DALYs increased from 58,637.23 to 105,554.89 among males and from 57,861.91 to 90,801.25 among females. Male DALY counts and ASDR point estimates were higher than the corresponding female estimates in both years; in 2023, the male and female ASDRs were 67.84 and 56.95 per 100,000 population, respectively (Table 2).

**Table 2:** DALY cases and ASDR per 100,000 population of MM among the elderly population across East Asian countries in 1990 and 2023.

Characteristics	1990		2023		1990-2023
	DALYs.Cases.No.(95% UI)	ASDR per 100,000 No.(95% UI)	DALYs.Cases.No.(95% UI)	ASDR per 100,000 No.(95% UI)	EAPC(%) in ASDR No.(95% CI)
Eastern Asia	116499.14 (78155.93,155712.94)	59.04 (39.8,82.01)	196356.14 (143764.79,283053.36)	62.45 (46.3,86.69)	47.81 (−126.51,299.89)
People’s Republic of China	98385.6 (63584.53,132734.14)	9.74 (6.36,13.17)	158132.8 (113814.49,233178.88)	7.57 (5.42,11.14)	13.05 (−7.6,36.84)
Japan	10580.44 (9120.2,12421.8)	6.65 (5.73,7.82)	19463.28 (16199.96,23209.66)	7.52 (6.47,8.91)	−1.25 (−24.32,32.32)
Republic of Korea	3720.79 (2551.4,5606.58)	9.87 (6.84,15.14)	8515.15 (6022.18,13283.84)	9.75 (6.95,15.19)	54.8 (2.22,127.07)
Democratic People’s Republic of Korea	1642.74 (1076.23,2378.75)	8.97 (5.83,12.85)	4633.75 (2916.44,6717.97)	13.88 (8.72,20.26)	−25.45 (−61.17,48.59)
Mongolia	156.93 (64.5,253.77)	12.81 (5.46,20.39)	277.12 (188.75,421.82)	9.55 (6.43,14.62)	29 (2.56,55.98)
Taiwan (Province of China)	2012.64 (1759.08,2317.89)	11 (9.59,12.64)	5334.05 (4622.96,6241.2)	14.19 (12.31,16.57)	−22.34 (−38.2,-0.9)
**Gender**					
Male	58637.23 (31148.11,89333.95)	62.86 (38.33,93.41)	105554.89 (66057.68,164358.59)	67.84 (45.04,101.38)	38.67 (−185.06,364.22)
Female	57861.91 (34132.14,96484.43)	55.53 (31.51,91.28)	90801.25 (54496.33,145893.09)	56.95 (36.44,89.88)	81.33 (−144.29,518.37)

### 3.2 Regional burden analysis

In 2023, the four age-standardized indicators of MM among the elderly population varied across the six included East Asian locations (Table 3). For both sexes combined, Taiwan (Province of China) had the highest ASIR at 2.79 per 100,000 population (95% UI: 2.02–3.68), followed by the Republic of Korea at 2.56 (95% UI: 1.64–4.29) and Japan at 2.14 (95% UI: 1.53–2.86). The corresponding ASIRs were lower in the Democratic People’s Republic of Korea at 1.25 (95% UI: 0.74–1.99), the People’s Republic of China at 0.79 (95% UI: 0.54–1.25), and Mongolia at 0.72 (95% UI: 0.47–1.19). A similar location ordering was observed for ASPR. Taiwan (Province of China) had the highest ASPR at 22.02 per 100,000 population (95% UI: 15.45–29.73), followed by the Republic of Korea at 20.39 (95% UI: 12.37–35.61) and Japan at 17.85 (95% UI: 12.60–23.82). Lower ASPR point estimates were observed in the Democratic People’s Republic of Korea at 7.43 (95% UI: 4.20–12.34), the People’s Republic of China at 5.03 (95% UI: 3.17–8.48), and Mongolia at 3.54 (95% UI: 1.95–6.54). Female ASIR and ASPR point estimates were higher than the corresponding male estimates in all six locations.

**Table 3:** Age-standardized rates of MM among the elderly population in six East Asian locations in 2023, by sex.

Location	ASIR in 2023 per 100,000 persons			ASPR in 2023 per 100,000 persons			ASMR in 2023 per 100,000 persons			ASDR in 2023 per 100,000 persons		
	Both	Male	Female	Both	Male	Female	Both	Male	Female	Both	Male	Female
**People’s Republic of China**	0.79(0.54,1.25)	0.76(0.44,1.24)	0.82(0.47,1.50)	5.03(3.17,8.48)	4.60(2.70,7.84)	5.42(3.00,10.27)	0.27(0.19,0.39)	0.28(0.16,0.44)	0.26(0.15,0.42)	7.57(5.42,11.14)	8.30(4.88,13.32)	6.75(3.89,11.19)
**Taiwan (Province of China)**	2.79(2.02,3.68)	2.69(1.92,3.65)	2.90(2.02,4.10)	22.02(15.45,29.73)	20.35(13.75,28.67)	23.66(16.38,33.45)	0.47(0.41,0.55)	0.55(0.46,0.67)	0.40(0.32,0.49)	14.19(12.31,16.57)	16.94(14.03,20.69)	11.60(9.50,14.31)
**Japan**	2.14(1.53,2.86)	1.95(1.37,2.65)	2.36(1.66,3.22)	17.85(12.60,23.82)	15.56(10.49,21.85)	20.29(13.93,27.65)	0.25(0.21,0.29)	0.30(0.25,0.37)	0.21(0.16,0.26)	7.52(6.47,8.91)	8.39(6.83,10.23)	6.79(5.41,8.54)
**Democratic People’s Republic of Korea**	1.25(0.74,1.99)	1.01(0.52,1.76)	1.42(0.64,2.57)	7.43(4.20,12.34)	5.36(2.62,9.60)	9.14(4.37,16.49)	0.47(0.29,0.69)	0.43(0.23,0.71)	0.48(0.26,0.84)	13.88(8.72,20.26)	13.98(7.40,22.61)	13.27(7.19,23.22)
**Republic of Korea**	2.56(1.64,4.29)	2.28(1.32,4.16)	2.88(1.55,5.50)	20.39(12.37,35.61)	17.60(10.26,32.48)	23.28(12.86,44.85)	0.34(0.24,0.50)	0.39(0.23,0.65)	0.30(0.17,0.50)	9.75(6.95,15.19)	10.63(6.43,18.16)	8.98(5.09,15.40)
**Mongolia**	0.72(0.47,1.19)	0.60(0.34,1.09)	0.81(0.46,1.55)	3.54(1.95,6.54)	1.78(0.91,3.44)	5.07(2.63,10.06)	0.37(0.24,0.57)	0.36(0.21,0.62)	0.37(0.20,0.64)	9.55(6.43,14.62)	9.59(5.47,16.38)	9.55(5.37,17.22)

The location pattern of fatal outcomes differed from that of incidence and prevalence. Taiwan (Province of China) and the Democratic People’s Republic of Korea had the highest ASMR point estimates, both at 0.47 per 100,000 population, with 95% UIs of 0.41–0.55 and 0.29–0.69, respectively. They were followed by Mongolia at 0.37 (95% UI: 0.24–0.57), the Republic of Korea at 0.34 (95% UI: 0.24–0.50), the People’s Republic of China at 0.27 (95% UI: 0.19–0.39), and Japan at 0.25 (95% UI: 0.21–0.29). Taiwan (Province of China) also had the highest ASDR at 14.19 per 100,000 population (95% UI: 12.31–16.57), followed by the Democratic People’s Republic of Korea at 13.88 (95% UI: 8.72–20.26), the Republic of Korea at 9.75 (95% UI: 6.95–15.19), Mongolia at 9.55 (95% UI: 6.43–14.62), the People’s Republic of China at 7.57 (95% UI: 5.42–11.14), and Japan at 7.52 (95% UI: 6.47–8.91). Male ASDR point estimates were higher than the corresponding female estimates in all six locations. Male ASMR point estimates were higher in the People’s Republic of China, Taiwan (Province of China), Japan, and the Republic of Korea, whereas female ASMR point estimates were slightly higher in the Democratic People’s Republic of Korea and Mongolia. The complete sex-specific estimates and their corresponding 95% UIs are presented in Table 3.

### 3.3 Age and sex differences in MM among the elderly population in East Asia in 2023

**Fig 1:**
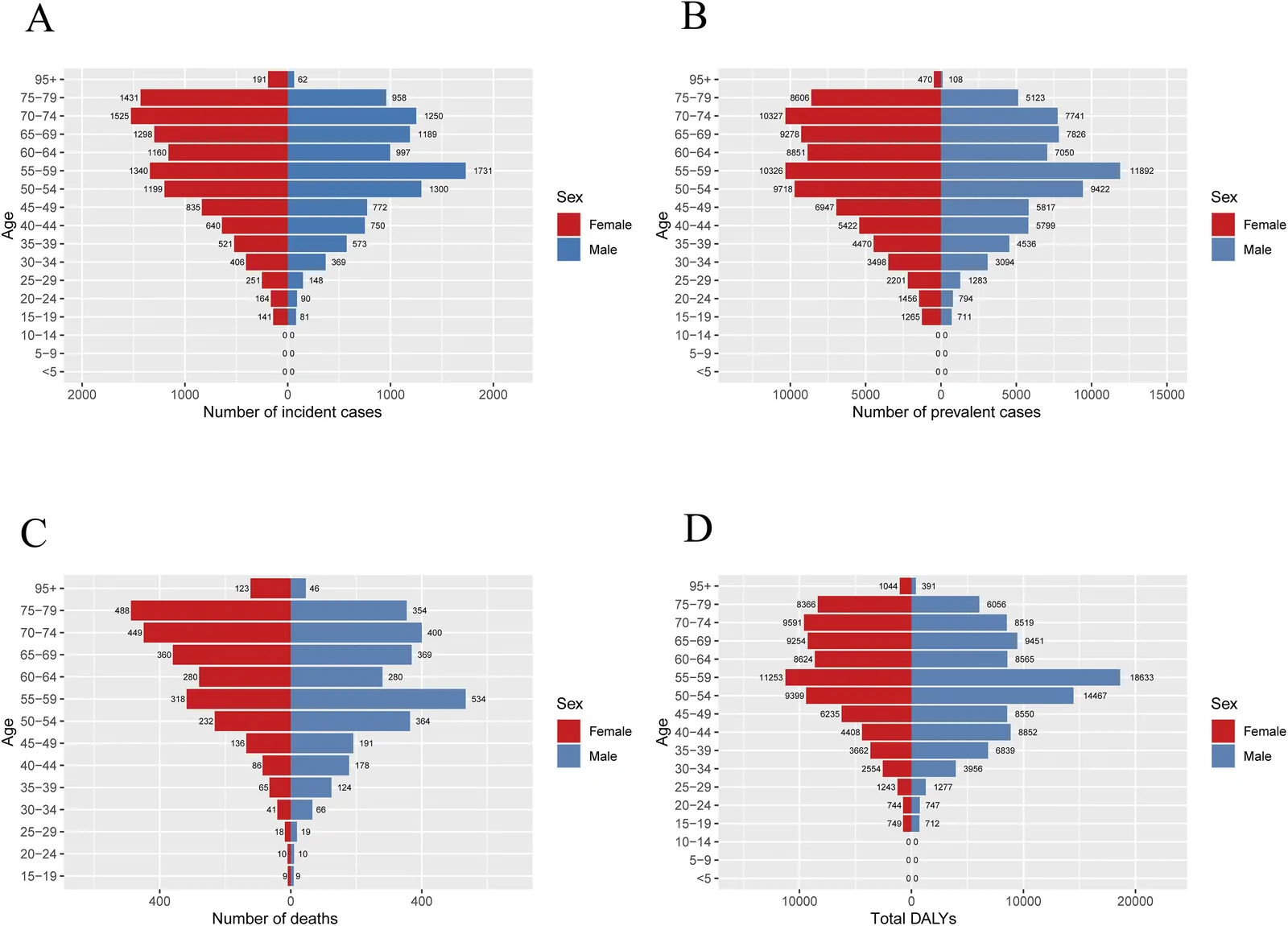
Age and sex differences in the absolute numbers of MM-related outcomes among the elderly population in East Asia in 2023. (A) Incident cases. (B) Prevalent cases. (C) Deaths. (D) DALYs.

The absolute numbers of MM-related outcomes showed non-monotonic age patterns that varied by sex and indicator in 2023 (Fig 1). When both sexes were combined, the numbers of incident cases, prevalent cases, deaths, and DALYs were all greatest in the 55–59-year age group, reaching 3,071, 22,218, 852, and 29,886, respectively; however, the numbers of deaths were very similar across the 55–59-, 70–74-, and 75–79-year age groups, at 852, 849, and 842, respectively. Among females, the numbers of incident and prevalent cases were greatest at ages 70–74, reaching 1,525 and 10,327, respectively, although the number of prevalent cases at ages 55–59 was nearly identical (10,326); female deaths were greatest at ages 75–79 (488), whereas female DALYs were greatest at ages 55–59 (11,253). Among males, all four outcomes reached their greatest absolute values at ages 55–59, including 1,731 incident cases, 11,892 prevalent cases, 534 deaths, and 18,633 DALYs. Overall, the distributions exhibited a prominent peak at ages 55–59 for males and later peaks for several female outcomes, rather than a uniform unimodal pattern across all indicators.

**Fig 2:**
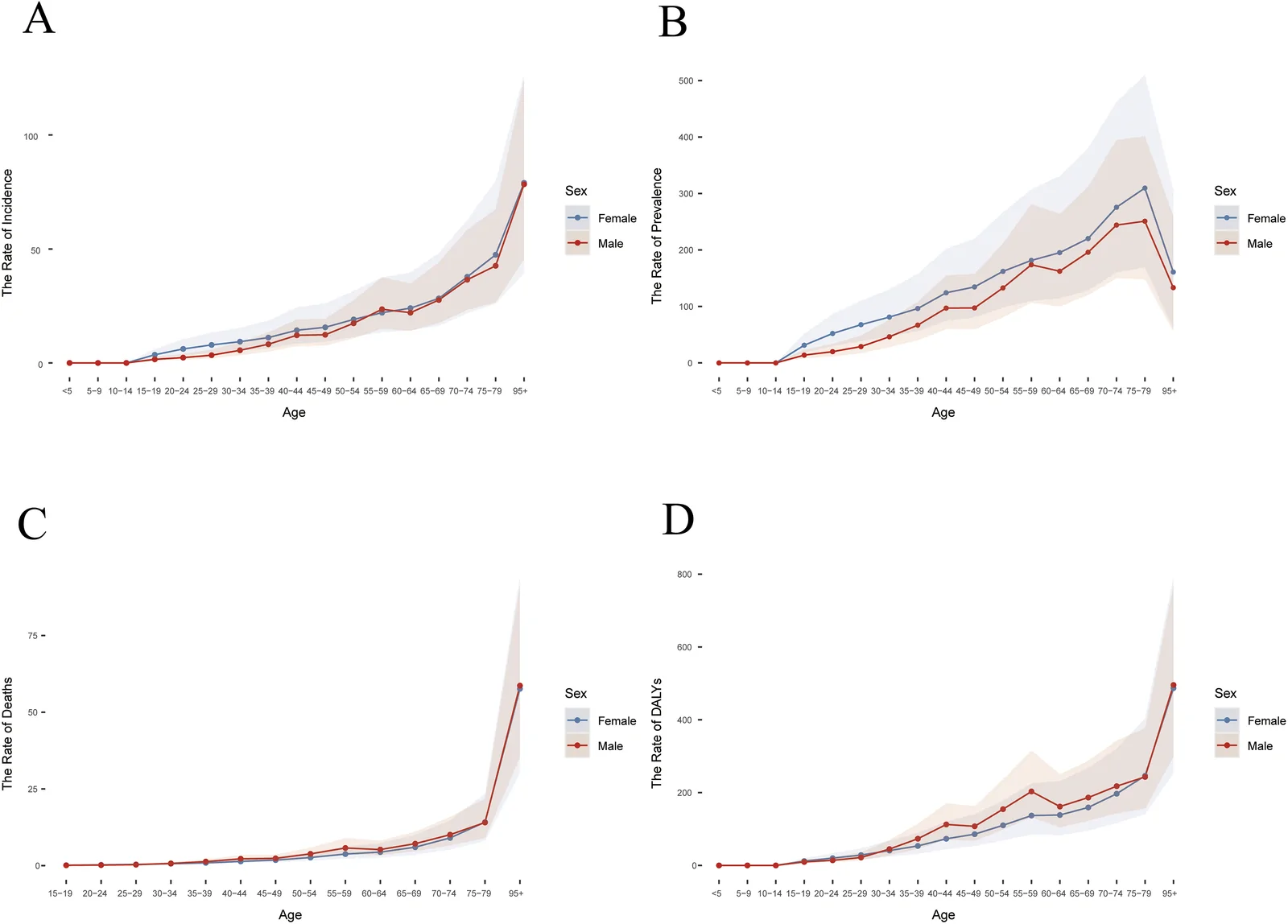
Age and sex differences in age-specific rates of MM among the elderly population in East Asia in 2023. (A) Incidence rates. (B) Prevalence rates. (C) Death rates. (D) DALY rates.

Unlike the absolute case counts, which did not increase consistently with age, age-specific rates generally increased with advancing age, although modest fluctuations were observed in several age groups (Fig 2). Incidence, mortality, and DALY rates reached their highest values in the 95 + age group for both sexes, with mortality and DALY rates increasing particularly sharply at the oldest ages. In contrast, the prevalence rate increased until the age of 75–79 years and subsequently declined markedly in the oldest age groups. Females generally had higher incidence and prevalence rates than males across most age groups. Mortality and DALY rates were broadly similar between the sexes at younger ages, whereas male rates were generally higher across many middle-aged and older age groups.

**Fig 3:**
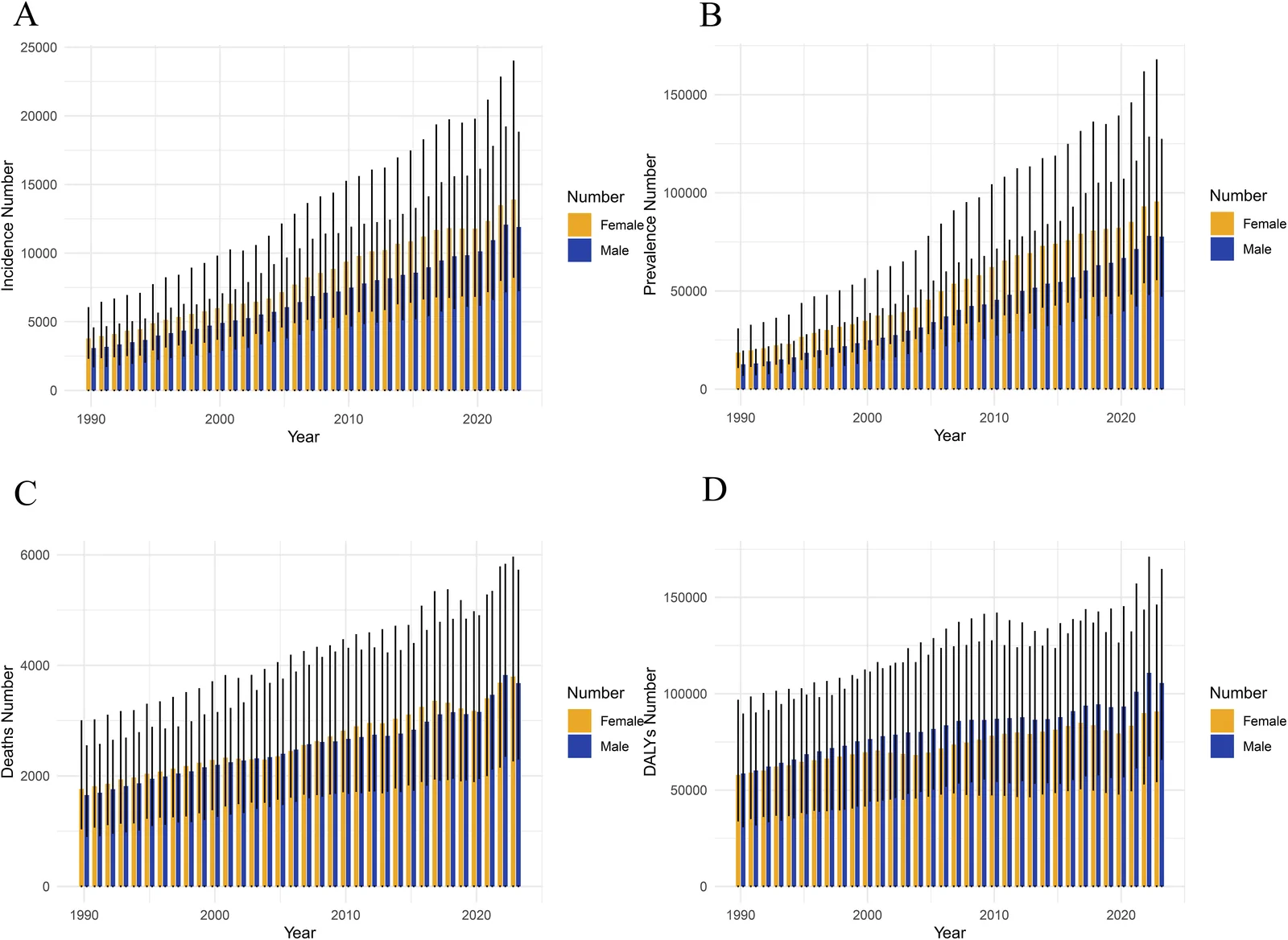
Temporal trends in the absolute numbers of MM-related outcomes among the elderly population in East Asia, 1990–2023. (A) Incident cases. (B) Prevalent cases. (C) Deaths. (D) DALYs.

**Fig 4:**
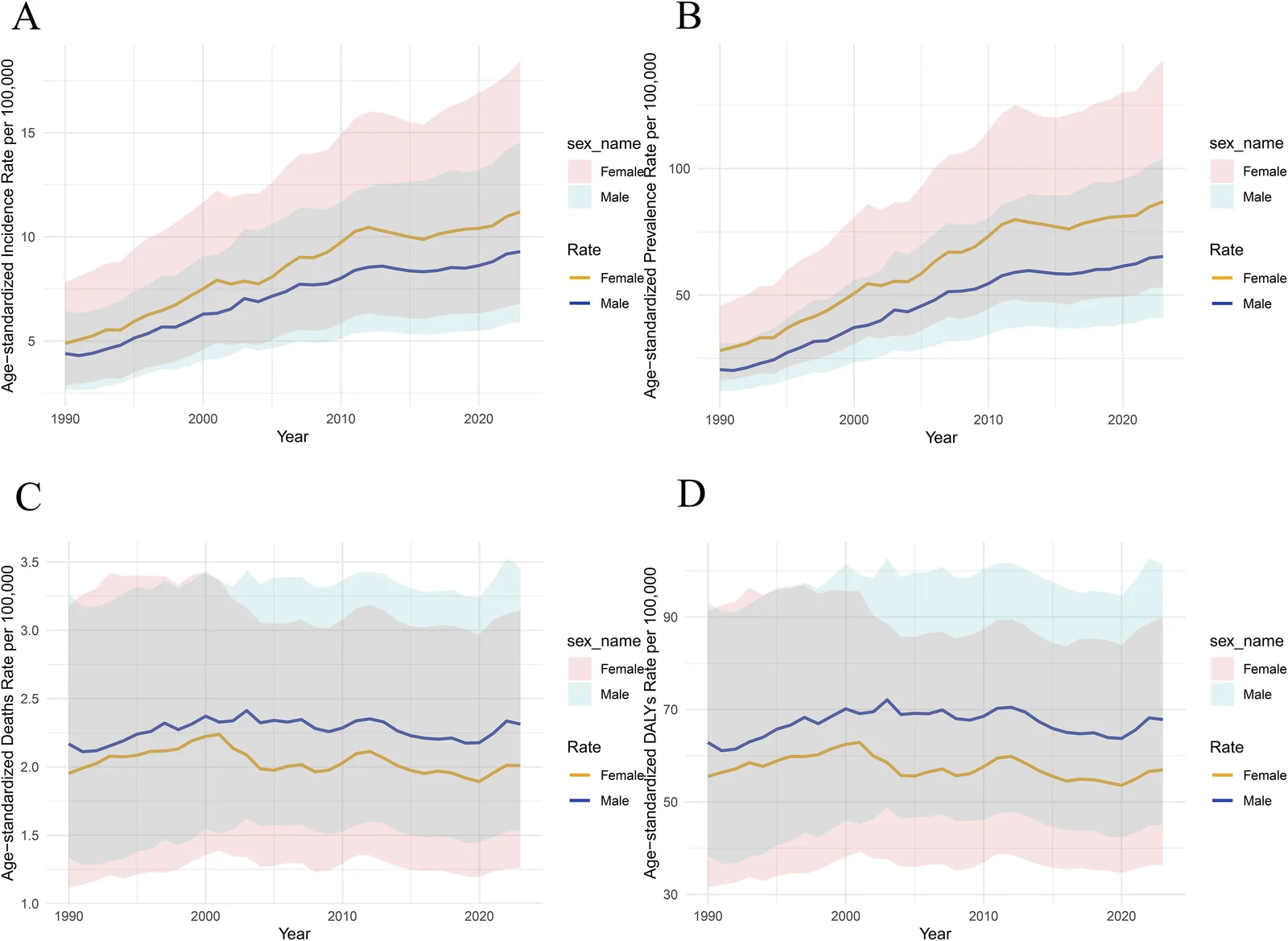
Temporal trends in age-standardized rates of MM among the elderly population in East Asia, 1990–2023. (A) ASIR. (B) ASPR. (C) ASMR. (D) ASDR.

Over the 34-year study period from 1990 to 2023, the absolute numbers of incident cases, prevalent cases, deaths, and DALYs attributable to MM generally increased in both sexes, although year-to-year fluctuations were observed (Figs 3 and 4). ASIR and ASPR also showed overall upward trends, with females consistently exhibiting higher rates than males throughout the study period. Although these trends were interrupted by occasional plateaus or modest declines, the relative sex difference in incidence and prevalence remained broadly stable. In contrast, ASMR and ASDR displayed fluctuating rather than steadily increasing trends over time. Male ASMR and ASDR remained higher than the corresponding female rates throughout the study period; however, the magnitude of the sex difference varied over time and did not show a consistent widening pattern.

### 3.4 Age-specific and regional patterns of MM rates among the elderly population in East Asia

**Fig 5:**
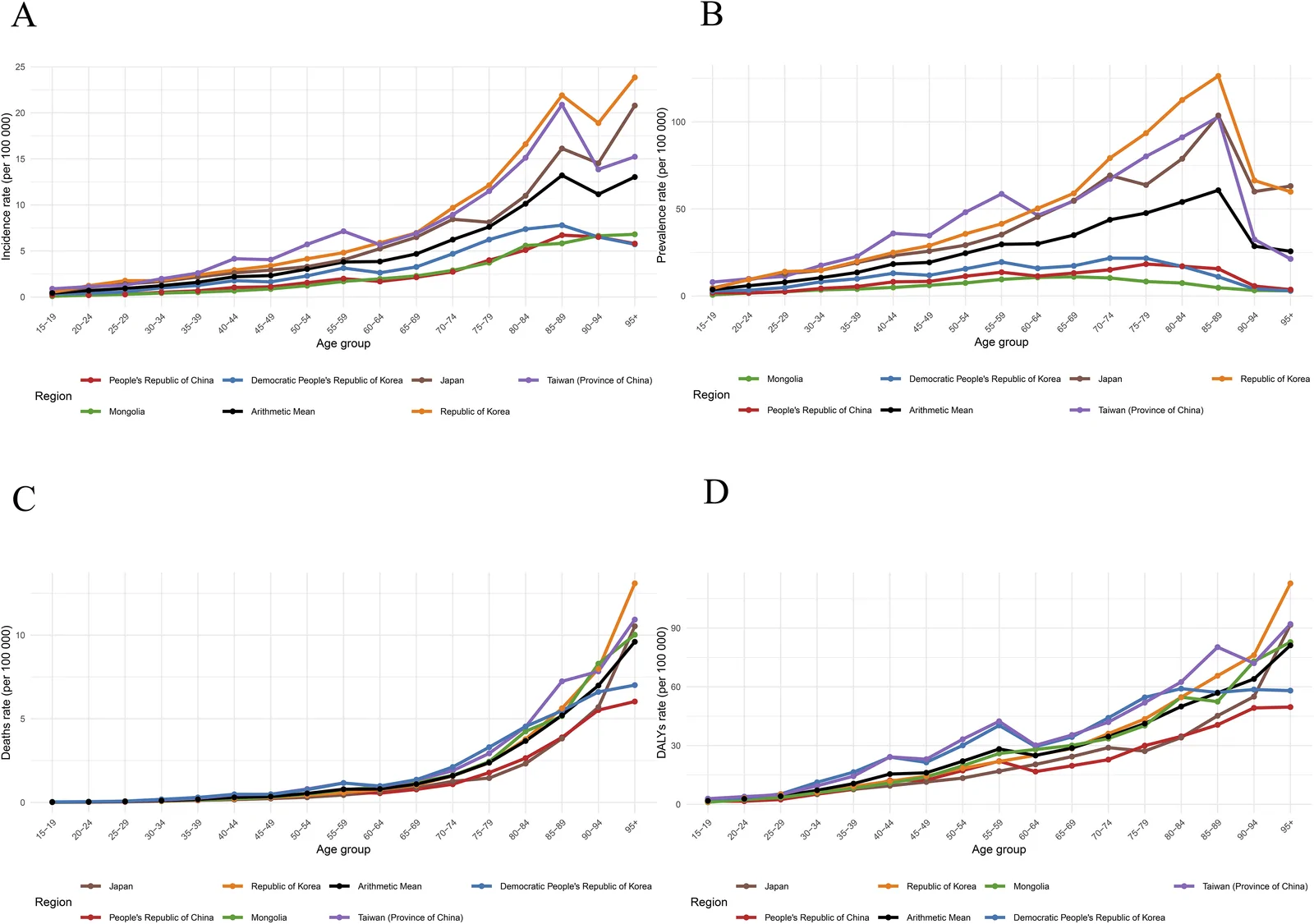
Age-specific and regional patterns of MM burden among the elderly population across East Asian countries in 2023. (A) Incidence rates.(B) Prevalence rates.(C) Death rates.(D) DALY rates.

In 2023, age-specific rates of MM varied substantially across age groups and locations in East Asia (Fig 5). Incidence rates generally increased with age in all six locations. The Republic of Korea showed the highest incidence rates across most older age groups, particularly after age 65, followed mainly by Taiwan (Province of China) and Japan. The People’s Republic of China, Mongolia, and the Democratic People’s Republic of Korea generally exhibited lower incidence rates. For prevalence, Taiwan (Province of China) showed comparatively high rates in several middle-aged groups, whereas the Republic of Korea generally had the highest rates from approximately age 60–94. Japan also exhibited relatively high prevalence rates in the oldest age groups and had the highest rate in the 95+ group, while China, Mongolia, and the Democratic People’s Republic of Korea generally remained at lower levels. Mortality rates were relatively low and similar across locations at younger ages, but regional differences became more evident after age 70. Taiwan (Province of China) and the Democratic People’s Republic of Korea showed comparatively high mortality rates in several age groups between 70 and 89 years, whereas the Republic of Korea experienced a particularly steep increase at the oldest ages and had the highest mortality rate in the 95+ group. Japan and Mongolia also showed relatively high mortality rates in this oldest group, while China and the Democratic People’s Republic of Korea had lower rates. A similar age-related divergence was observed for DALY rates. Taiwan (Province of China) and the Democratic People’s Republic of Korea exhibited comparatively high DALY rates in several middle-aged and early older age groups. At more advanced ages, the rates increased markedly in the Republic of Korea, Taiwan (Province of China), Mongolia, and Japan, with the Republic of Korea reaching the highest DALY rate in the 95+ group. In contrast, China and the Democratic People’s Republic of Korea showed comparatively lower DALY rates in the oldest age group. Overall, the rankings of the six locations were not uniform but varied according to the epidemiological indicator and age group.

### 3.5 Joinpoint regression analysis of age-standardized MM rates

**Fig 6:**
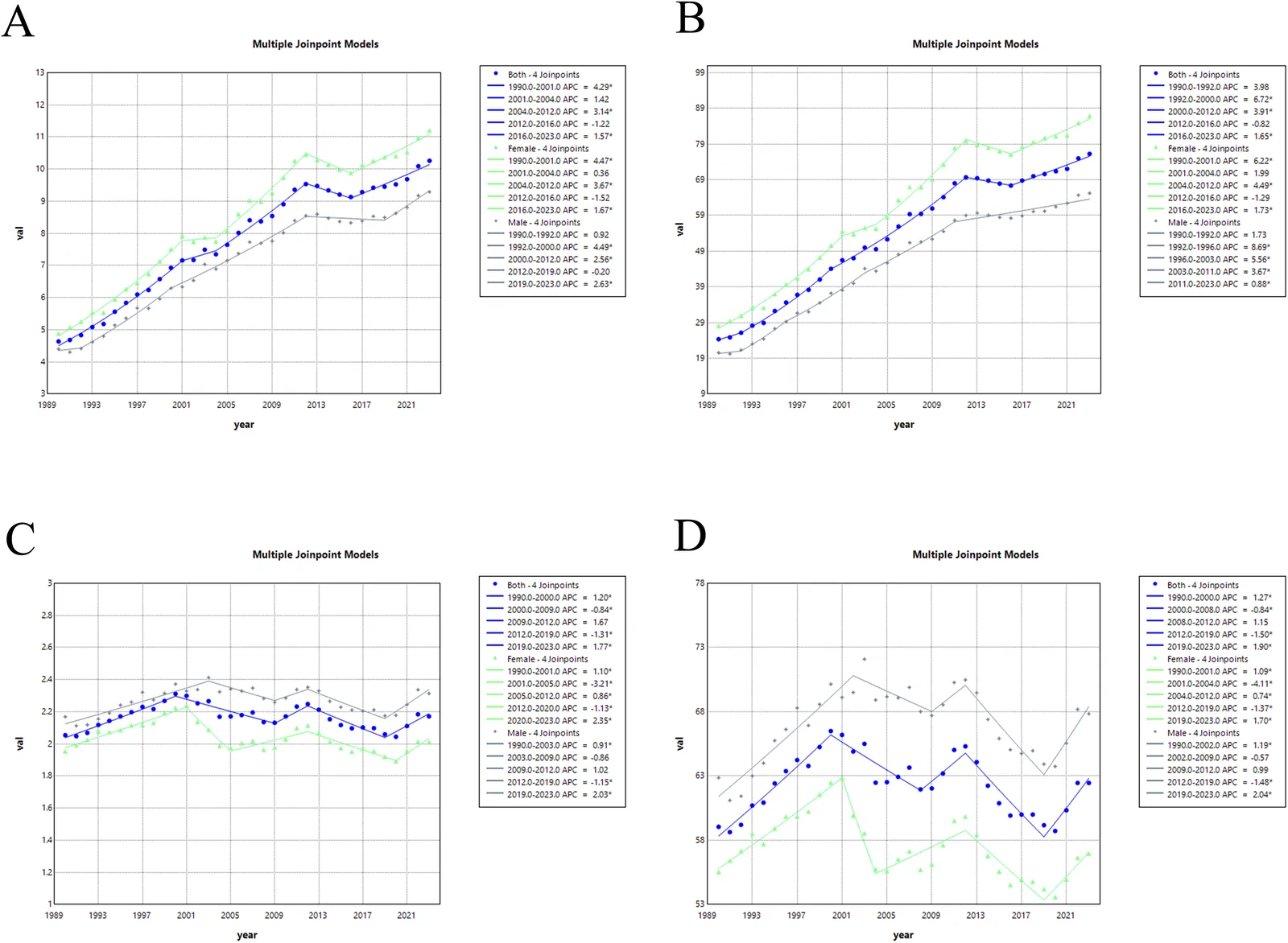
Joinpoint regression analysis of MM burden temporal trends among the elderly population across East Asian countries, 1990–2023. (A) ASIR.(B) ASPR.(C) ASMR.(D) ASDR.

Joinpoint regression revealed segmented temporal patterns in the burden of MM among the older population in East Asia from 1990 to 2023 (Fig 6A–6D). ASIR increased significantly overall (AAPC = 2.49%; 95% CI: 2.01%–2.98%; p < 0.001). It increased significantly during 1990–2001 (APC = 4.29%; 95% CI: 3.99%–4.60%; p < 0.001), showed a non-significant increase during 2001–2004 (APC = 1.42%; 95% CI: −2.63%–5.65%; p = 0.478), increased significantly during 2004–2012 (APC = 3.14%; 95% CI: 2.55%–3.73%; p < 0.001), declined non-significantly during 2012–2016 (APC = −1.22%; 95% CI: −3.34%–0.96%; p = 0.255), and increased significantly again during 2016–2023 (APC = 1.57%; 95% CI: 0.93%–2.21%; p < 0.001). ASPR showed the largest significant overall increase (AAPC = 3.61%; 95% CI: 3.22%–4.00%; p < 0.001), with significant increases during 1990–2000 (APC = 6.44%; 95% CI: 5.98%–6.90%; p < 0.001) and 2000–2012 (APC = 3.96%; 95% CI: 3.59%–4.33%; p < 0.001), a non-significant decline during 2012–2016 (APC = −0.87%; 95% CI: −3.41%–1.73%; p = 0.492), and a significant increase during 2016–2023 (APC = 1.66%; 95% CI: 0.91%–2.41%; p < 0.001). In contrast, ASMR (AAPC = 0.23%; 95% CI: −0.21%–0.67%; p = 0.307) and ASDR (AAPC = 0.23%; 95% CI: −0.24%–0.71%; p = 0.336) showed small overall changes. Both rates increased significantly during 1990–2000, declined significantly during 2000–2009, displayed non-significant fluctuations during the intermediate periods, and increased significantly again during 2020–2023 (ASMR: APC = 2.16%; 95% CI: 0.46%–3.89%; p = 0.015; ASDR: APC = 2.22%; 95% CI: 0.36%–4.12%; p = 0.022). Female incidence and prevalence rates remained higher than the corresponding male rates, and both sexes experienced significant long-term increases in these morbidity indicators. Conversely, mortality and DALY rates remained higher in males. The long-term increases in ASMR and ASDR were statistically significant among males (ASMR: AAPC = 0.30%; 95% CI: 0.05%–0.55%; p = 0.018; ASDR: AAPC = 0.34%; 95% CI: 0.06%–0.62%; p = 0.019) but not among females. Overall, the age-standardized morbidity burden of MM increased significantly, despite temporary interruptions.

### 3.6 Results of the age-period-cohort analysis

**Fig 7:**
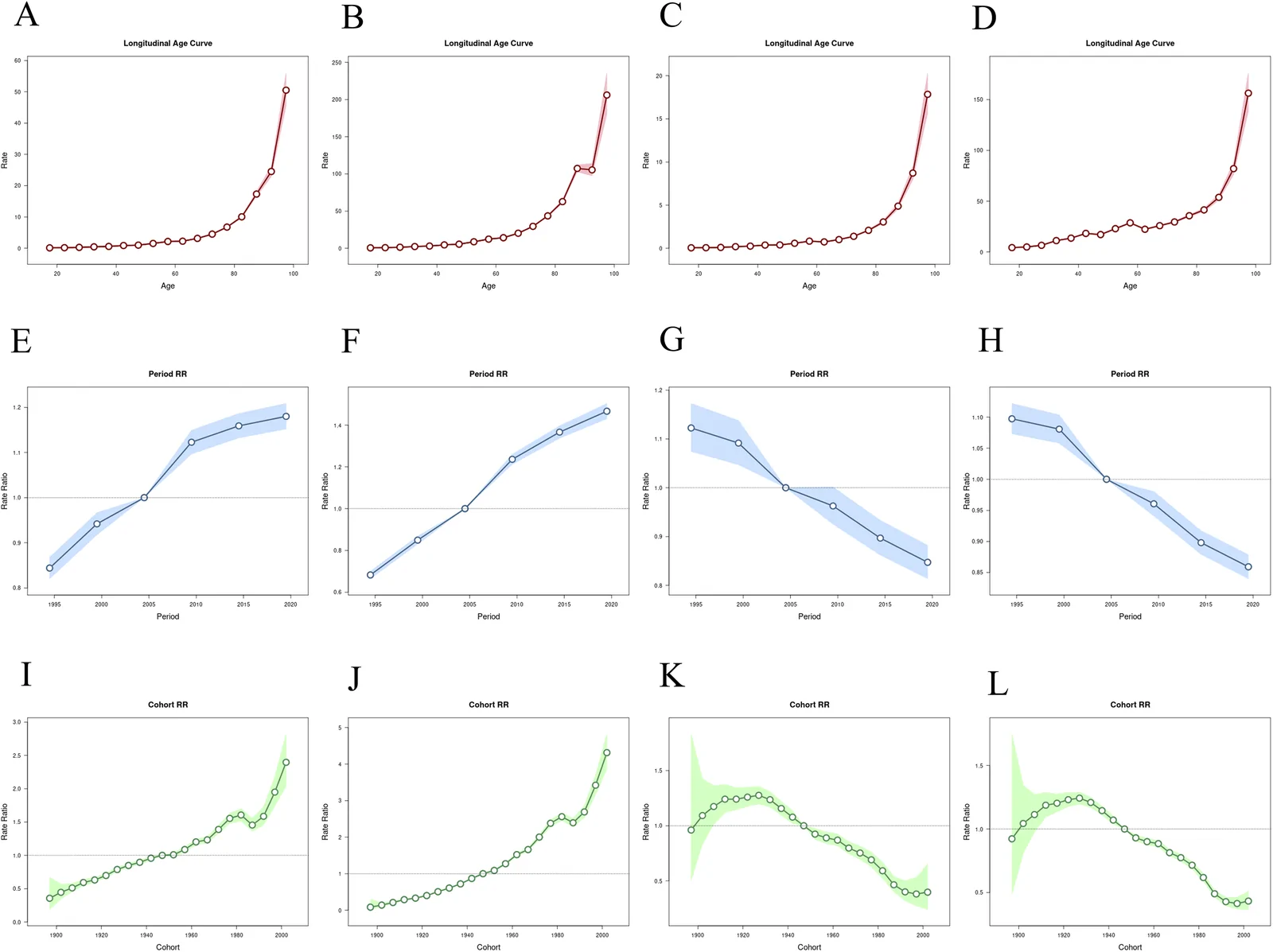
Age–period–cohort analysis results for MM burden among the elderly population across East Asian countries, 1990–2023. (A) Longitudinal age curve for incidence. (B) Longitudinal age curve for prevalence. (C) Longitudinal age curve for deaths. (D) Longitudinal age curve for DALYs. (E) Period RR for incidence. (F) Period RR for prevalence. (G) Period RR for deaths. (H) Period RR for DALYs. (I) Cohort RR for incidence. (J) Cohort RR for prevalence. (K) Cohort RR for deaths. (L) Cohort RR for DALYs.

The age–period–cohort (APC) analysis demonstrated distinct age, period, and birth-cohort patterns in the burden of MM in East Asia from 1990 to 2023 (Fig 7A–7L). The longitudinal age curves showed that incidence, prevalence, mortality, and DALY rates generally increased with age, although modest fluctuations were observed in the prevalence and DALY curves. The increases became more pronounced in older age groups, particularly after approximately 80 years, and all four indicators reached their highest levels in the oldest age group (Fig 7A–7D), indicating a strong association between advancing age and MM burden. The period rate ratios for incidence and prevalence increased across successive periods, whereas those for mortality and DALYs declined over time (Fig 7E–7H). The cohort rate ratios for incidence and prevalence generally increased among later birth cohorts, although the trajectories were not completely monotonic (Fig 7I and 7J). In contrast, the cohort rate ratios for mortality and DALYs initially increased among the earlier birth cohorts, reached relatively high levels among cohorts born around the 1920s, and subsequently showed an overall decline among later birth cohorts, with some fluctuations at the extremes of the cohort range (Fig 7K and 7L). Overall, the APC analysis identified a pronounced age gradient and opposing period and cohort patterns between morbidity and fatal outcomes.

### 3.7 Future projections

**Fig 8:**
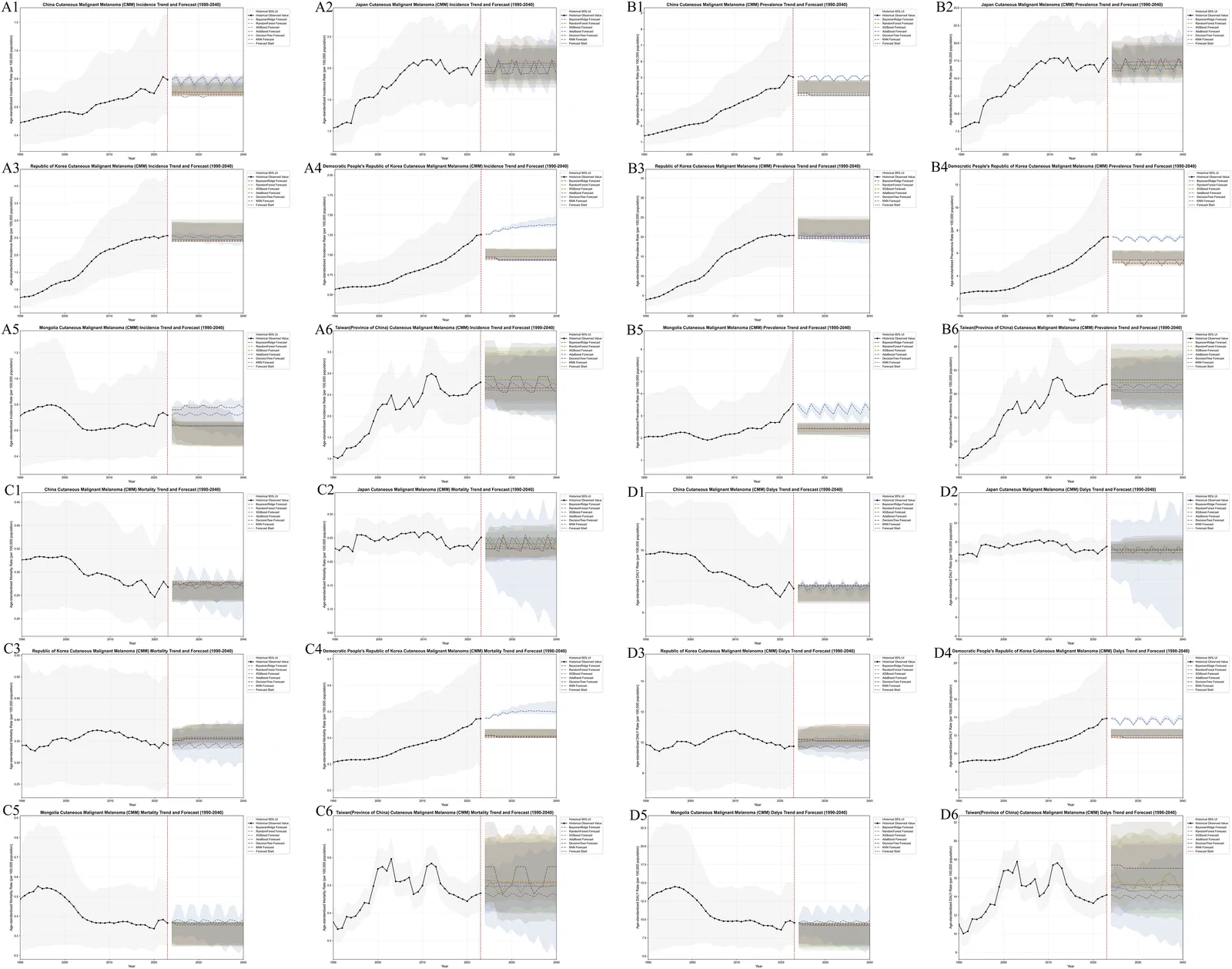
Future projections to 2040 for MM in the People’s Republic of China, Japan, the Republic of Korea, the Democratic People’s Republic of Korea, Mongolia, and Taiwan (Province of China). (A1-A6) Incidence; (B1-B6) Prevalence; (C1-C6) Mortality; (D1-D6) DALYs.

Based on observations through 2023, the burden of MM in six East Asian locations was projected from 2024 to 2040 using the multi-model forecasting framework. For each location–indicator series, the model selected through rolling time-series cross-validation was used to report the point forecast and residual-bootstrap 95% prediction interval (PI) (Fig 8).

#### 3.7.1 Projections for ASIR and ASPR.

ASIR and ASPR were projected to remain broadly stable, although modest location-specific increases and decreases were observed. By 2040, Taiwan (Province of China) was projected to have the highest ASIR at 2.7274 per 100,000 population (95% PI: 1.7869–3.6244), followed by the Republic of Korea at 2.5418 (95% PI: 2.2872–2.6002) and Japan at 2.0312 (95% PI: 1.7166–2.4798). The corresponding projected ASIR was 1.3802 in the Democratic People’s Republic of Korea (95% PI: 1.3539–1.4849), 0.8140 in China (95% PI: 0.7539–0.8387), and 0.7365 in Mongolia (95% PI: 0.5671–0.8631).

A similar regional ordering was observed for ASPR. Taiwan (Province of China) was projected to have the highest ASPR in 2040 at 21.8468 per 100,000 population (95% PI: 14.4764–28.5963), followed by the Republic of Korea at 20.3424 (95% PI: 18.3530–20.8056) and Japan at 17.0479 (95% PI: 15.8709–20.8979). The projected ASPR was 7.3677 in the Democratic People’s Republic of Korea (95% PI: 7.2537–7.5766), 5.1073 in China (95% PI: 5.0698–5.1536), and 3.2563 in Mongolia (95% PI: 2.7901–3.4726).

#### 3.7.2 Projections for ASMR and ASDR.

ASMR and ASDR were projected to remain broadly stable or change only modestly through 2040, although the direction and magnitude of change varied by location. The Democratic People’s Republic of Korea was projected to have the highest ASMR in 2040 at 0.4992 per 100,000 population (95% PI: 0.4901–0.5405), followed by Taiwan (Province of China) at 0.4647 (95% PI: 0.2562–0.6407). The projected ASMR was 0.3824 in Mongolia (95% PI: 0.2410–0.4444), 0.3452 in the Republic of Korea (95% PI: 0.2990–0.4033), 0.2790 in China (95% PI: 0.2102–0.3047), and 0.2306 in Japan (95% PI: 0.2090–0.2504).

For ASDR, Taiwan (Province of China) and the Democratic People’s Republic of Korea were projected to have the highest rates in 2040, at 14.0074 (95% PI: 7.9589–18.8934) and 13.7805 (95% PI: 13.6451–14.1573) per 100,000 population, respectively. The projected ASDR was 9.8306 in Mongolia (95% PI: 6.1238–12.0736), 9.7478 in the Republic of Korea (95% PI: 8.8921–10.5918), 7.9398 in China (95% PI: 7.5058–8.1220), and 7.1005 in Japan (95% PI: 6.3602–8.1678). Overall, these projections suggest that age-standardized morbidity and fatal-outcome rates will remain heterogeneous across East Asia but will generally change only modestly through 2040. The projections should be interpreted cautiously because they extrapolate historical patterns and do not account for unforeseen changes in diagnosis, treatment, or healthcare policy.

## 4. Discussion

This study represents the first elderly-specific analysis of long-term MM trends across East Asia, highlighting population aging as a central demographic context for understanding the evolving disease burden [18]. Despite only modest changes in the age-standardized mortality and DALY rates between 1990 and 2023 (ASMR: 2.05 to 2.17; ASDR: 59.04 to 62.45 per 100,000), East Asia experienced a substantial increase in the absolute number of melanoma-related deaths (from 3,417.59 to 7,476.55) and DALYs (from 116,499.14 to 196,356.14) among the elderly. This divergence suggests that demographic aging, together with growth in the older population, has made an important contribution to the increasing absolute burden, rather than the increase being attributable solely to changes in age-standardized fatal-outcome rates [19]. This pattern is broadly consistent with global observations but is particularly relevant to East Asia because the region is undergoing one of the world’s most rapid and largest transitions toward an older population structure [20].

When the findings are compared with global or Western regions, East Asia demonstrates a distinct epidemiological and clinical pattern [21]. Western countries, including those in Europe, Australia, and North America, report substantially higher melanoma incidence, driven primarily by fair skin phenotypes and greater intermittent UV exposure [22,23]. However, the clinical profile of melanoma in East Asia is markedly different. ALM and mucosal melanoma account for a greater proportion of cases in East Asian populations than in Western populations [24]. ALM typically occurs on the palms, soles, and nail beds, whereas mucosal melanoma arises on mucosal surfaces; both subtypes are less strongly associated with UV exposure than conventional cutaneous melanoma [25]. This distinct subtype distribution may partly contribute to the relatively low overall incidence but substantial fatal burden of melanoma among older adults in East Asia. In particular, ALM can be difficult to detect at an early stage because of its less-visible anatomical locations and subtle initial manifestations, potentially leading to delayed diagnosis and more advanced disease at the time of identification [26].

Age-specific results from this study further demonstrate that MM rates generally rise steeply with advancing age across East Asian settings [27]. Although the age-specific patterns of incidence and prevalence varied by location, mortality and DALY rates generally continued to increase into the oldest age groups and reached their highest levels among individuals aged 95 years or older. The timing and magnitude of the observed peaks differed across locations and epidemiological indicators, reflecting heterogeneity in population structure, disease detection, and healthcare access. This pronounced age-associated increase is biologically and epidemiologically plausible, as immunosenescence, accumulated lifetime exposures, reduced attention to skin changes, and delayed diagnosis may contribute to poorer melanoma outcomes in older age groups [28]. Importantly, the prominent age gradient observed in this study underscores the particular relevance of demographic aging to melanoma prevention and healthcare planning in East Asia [29]. As Japan, the Republic of Korea, China, and Taiwan (Province of China) continue to experience rapid population aging and expansion of their older populations, the demand for melanoma detection, treatment, and long-term care among older adults is likely to increase further [30].

Notable sex differences were identified in the patterns of MM outcomes [31]. In 2023, men had a higher age-standardized mortality-rate point estimate of 2.31 per 100,000 than women at 2.01 per 100,000 and also exhibited a higher age-standardized DALY rate. Although the uncertainty intervals overlapped, the persistence of higher male mortality and DALY-rate estimates across multiple analyses indicates an epidemiologically relevant sex-specific pattern. This direction is broadly consistent with global observations, and behavioral and social differences in health awareness and healthcare utilization may contribute to the disparity [32]. Given the relatively high proportion of ALM in East Asian populations, delayed recognition of less-visible lesions on the soles, palms, or nail beds may be particularly important. Older men may be less likely to routinely examine these anatomical areas and may delay seeking medical evaluation for suspicious pigmented lesions, whereas older women may participate more frequently in routine health examinations and pay closer attention to physical changes in their extremities [33]. Nevertheless, women also experienced marked increases in DALY rates at advanced ages, indicating that any potential behavioral advantages do not eliminate the strong effect of aging on severe melanoma outcomes. These findings emphasize the need for sex-sensitive early-detection strategies, including greater awareness and clinical examination of the hands, feet, and nail beds, particularly among older men with persistently higher melanoma mortality-rate estimates [34].

Marked regional variation also shapes the melanoma burden among older adults in East Asia. In 2023, Taiwan (Province of China) and the Democratic People’s Republic of Korea had the highest point estimates for age-standardized mortality and DALY rates. China contributed the largest absolute number of deaths and DALYs, consistent with its large and rapidly aging population. Because ALM accounts for a relatively high proportion of melanoma cases in East Asian populations, these geographical variations are unlikely to be explained by climate or ultraviolet exposure alone. Instead, they may also be related to differences in health literacy, diagnostic capacity, cancer-registry coverage, access to specialized dermatological services, and the availability of advanced treatments. For instance, dermatological resources remain unevenly distributed across China, particularly between urban centers and rural or inland areas, which may contribute to delayed recognition of less-visible ALM lesions among older adults. The Democratic People’s Republic of Korea and Mongolia had comparatively low absolute numbers because of their smaller populations; however, their estimates should be interpreted cautiously because limited diagnostic and reporting capacity may affect case ascertainment. Compared with high-income Western countries with more established skin-surveillance systems and broader access to advanced therapies, the persistence of regional disparities in East Asia highlights the potential influence of healthcare-system capacity, diagnostic coverage, and equitable access to treatment on melanoma outcomes among older populations [8].

The age–period–cohort framework provides important insight into the temporal and generational patterns underlying these epidemiological trends. The longitudinal age curves show steep increases in incidence, prevalence, mortality, and DALY rates with advancing age, confirming a strong age-associated pattern in MM outcomes [35]. Period effects demonstrate continuous increases in incidence and prevalence, which may partly reflect improvements in diagnostic technologies, greater disease awareness, and expansion of cancer-registry coverage. In contrast, the period rate ratios for mortality and DALYs declined across the study period. Improvements in clinical management, including the introduction of immune checkpoint inhibitors and targeted therapies, may have contributed to more favorable fatal-outcome patterns in recent years; however, because the decline began before these therapies became widely available, it cannot be attributed solely to treatment advances. Cohort effects show that later birth cohorts had progressively higher relative risks of incidence and prevalence but lower relative risks of mortality and DALYs. These contrasting patterns may reflect generational differences in disease detection, healthcare access, environmental or occupational exposures, and clinical management. Nevertheless, because APC effects are descriptive population-level estimates, they should not be interpreted as identifying any single causal mechanism. The convergence of strong age, period, and cohort patterns highlights that continued population aging will remain a central demographic influence on the melanoma burden in East Asia over the coming decades.

Rapid population aging is central to interpreting the findings of this study. East Asia is undergoing one of the most accelerated demographic transitions in the world [36]. Population aging is particularly pronounced across the region: the proportion of residents aged 65 years or older in Japan is approaching 30%, while the Republic of Korea, Taiwan (Province of China), and China are also experiencing rapid growth in both the number and proportion of older adults [37]. Since melanoma is strongly age-related, expansion of the older population can produce substantial increases in the absolute disease burden even when age-standardized mortality and DALY rates change only modestly. When combined with the uneven distribution of specialized dermatological resources, limited elderly-focused early-detection programs, and variable access to advanced therapies, demographic aging may further magnify the vulnerability of older adults in East Asia [26]. Western countries also face population aging, but their longer-established melanoma awareness campaigns, dermatological diagnostic networks, and broader access to advanced therapies may partly mitigate its effect on melanoma mortality [38]. Comparable systems remain unevenly developed across East Asia, potentially increasing regional disparities in melanoma outcomes among older adults.

Several limitations must be acknowledged in this study. First, this study was a secondary analysis of modeled and aggregated estimates obtained from the GBD 2023 database rather than an analysis of primary cancer-registry or patient-level data. Therefore, the findings inevitably inherit uncertainties associated with the original data sources, differences in diagnostic practices and reporting quality, incomplete registry coverage, and the modeling procedures used by the GBD study. Second, our analysis was intentionally restricted to adults aged 60 years and older because the study specifically focused on the burden of malignant melanoma in East Asia’s aging population. Consequently, the findings should not be generalized to younger populations, and the exclusion of these populations prevents a comprehensive assessment of melanoma patterns across the entire age spectrum. Third, the GBD 2023 dataset does not stratify melanoma by specific histological subtypes, such as separating cutaneous melanoma from acral lentiginous melanoma (ALM) or mucosal melanoma. Given that ALM is the predominant subtype in East Asian populations, the inability to analyze its specific disease burden and distinct etiological drivers independently represents a major limitation of this research. Finally, although the GBD methodology is robust, estimates for regions with developing healthcare infrastructure, such as Mongolia and Democratic People’s Republic of Korea, may be influenced by limited local diagnostic capacity and incomplete cancer-registry coverage. These limitations should be considered when interpreting and generalizing the findings of this study.

In light of these distinct clinical characteristics, it becomes imperative to design and implement melanoma prevention strategies that are specifically tailored to the East Asian demographic [39]. Because ALM and mucosal types dominate the regional disease burden, relying primarily on Western-centric recommendations such as sun protection is unlikely to be sufficient for this specific population [8]. Priority actions should include expanding early detection through targeted screening of older adults, with particular attention to the hands, feet, and nail beds [40]. Public health campaigns should also promote routine self-examination of these anatomically concealed sites. Furthermore, healthcare systems should strengthen dermatologic capacity in underserved regions and improve equitable access to modern immunotherapies, thereby enabling older patients to receive timely diagnosis and appropriate treatment. These strategies should initially be implemented and assessed through prospective pilot programs. Future intervention evaluations should examine screening participation and coverage, referral and diagnostic yield, stage distribution at diagnosis, treatment uptake, patient outcomes, and healthcare-resource requirements. Formal health-economic studies should also evaluate the cost-effectiveness and feasibility of these interventions before their large-scale implementation.

## 5. Conclusion

From 1990 to 2023, absolute deaths and DALYs from MM increased substantially among older adults in East Asia, while age-standardized incidence and prevalence rates also showed significant long-term increases. Women had higher incidence and prevalence rates, whereas men had higher mortality and DALY rates. Although mortality and DALY rates may stabilize in some locations by 2040, these projections remain uncertain. Region- and sex-sensitive strategies should therefore strengthen early detection, examination of acral sites, and equitable access to treatment. Future research should develop subtype-specific cancer registries to independently evaluate the burden and etiology of ALM in East Asian populations.

## Supporting information

S1 Data Minimal dataset underlying the analyses of malignant skin melanoma incidence, prevalence, mortality, and DALYs in six East Asian locations from 1990 to 2023. (CSV)
