# Supplementary material for: Burden of malignant melanoma among the elderly population in East Asia from 1990 to 2023 and machine learning projection to 2040
Source: PLoS One. 2026 Sep 11;21(9):e0357767. doi: 10.1371/journal.pone.0357767 (PMC13568703; doi:10.1371/journal.pone.0357767)
Supplement: Supplementary file 1 [file pone.0357767.s002.docx]

**Response to Reviewers**

Manuscript Title: Burden of malignant melanoma among the elderly population in East Asia from 1990 to 2023 and machine learning projection to 2040

Journal: PLOS ONE

Submission Date of Revision: 2026.08.05

**General Response**

**Dear Academic Editor and Reviewers,**

Thank you for your careful evaluation of our manuscript and constructive comments. In response, we thoroughly cross-checked the revised text against the numerical results and figure legends, corrected inconsistencies between absolute counts and rates, clarified the methodology, data sources, and data accessibility, and expanded the discussion of study limitations and public health implications. For clarity, the reviewers’ comments are presented in italicized dark-gray text, our responses follow the green “Response:” label, and representative revisions to the manuscript are highlighted in yellow. We provide our point-by-point responses below.

（Note on figure renumbering: Following the removal of the original Figure 1 and its replacement with Table 3, the original Figures 2 and 3 have been renumbered as Figures 1 and 2, respectively. The original combined Figure 4 has been divided into revised Figures 3 and 4. Figures 5–8 retain their original numbering. The figure numbers in the reviewers’ original comments have been preserved unchanged, whereas all references in our responses and descriptions of manuscript revisions refer to the revised figure numbering.）

**REVIEWER #1**

**Comments on Bias, Limitations, Public Health, and Reproducibility**

**Comment 1.1:** *Please discuss potential biases in data collection, reporting, and projections, and explain their implications for the findings.*

**Response:** Thank you for emphasizing this important issue. We agree that the potential biases

and uncertainties associated with data collection, reporting, and forecasting should be explicitly acknowledged, together with their implications for the interpretation of our findings. First, this study is a secondary analysis of modeled and aggregated GBD 2023 estimates rather than primary cancer-registry or patient-level data. The findings therefore inevitably inherit uncertainties arising from the original data sources, regional differences in diagnostic practices and reporting quality, incomplete registry coverage, and the modeling procedures used by the GBD study. Second, the GBD 2023 dataset does not provide estimates stratified by melanoma histological subtype. This is particularly relevant in East Asia, where acral lentiginous melanoma (ALM) is the predominant subtype, and limits our ability to evaluate its specific burden and etiological characteristics independently. Third, estimates for regions with developing healthcare and surveillance systems, particularly Mongolia and the Democratic People’s Republic of Korea, may be affected by limited diagnostic capacity and incomplete cancer-registry coverage. These factors may result in under-ascertainment or differential

reporting and should be considered when interpreting cross-regional comparisons. In addition, because our analysis was restricted to adults aged 60 years and older, the findings should not be generalized to younger populations. Finally, the projections are based on historical trends and may not fully capture future changes in diagnostic practices, treatment availability,

healthcare policies, or other unforeseen factors. To reduce forecasting uncertainty and prevent data leakage, model selection was performed using rolling time-series cross-validation within the training set, and prediction intervals were generated using a residual-bootstrap procedure. Nevertheless, these procedures cannot eliminate all structural uncertainty; therefore, the projected estimates should be interpreted as plausible future trends rather than deterministic forecasts.

**Changes made in the manuscript (Methods, Sections 2.3.4-2.3.5; Discussion, Limitations):** *The revised Methods describes the chronological train-test split, rolling time-series cross-validation, model-selection procedure, and residual-bootstrap approach used to generate prediction intervals. The Limitations section has also been expanded to address uncertainties arising from the use of secondary modeled data, the exclusion of younger populations, the absence of histological-subtype stratification, incomplete diagnostic and registry coverage, and the extrapolation of historical trends:“Several limitations must be acknowledged in this study. First, this study was a secondary analysis of modeled and aggregated estimates obtained from the GBD 2023 database rather than an analysis of primary cancer-registry or patient-level data. Therefore, the findings inevitably inherit uncertainties associated with the original data sources, differences in diagnostic practices and reporting quality, incomplete registry coverage, and the modeling procedures used by the GBD study. Second, our analysis was intentionally restricted to adults aged 60 years and older because the study specifically focused on the burden of malignant melanoma in East Asia’s aging population. Consequently, the findings should not be generalized to younger populations, and the exclusion of these populations prevents a comprehensive assessment of melanoma patterns across the entire age spectrum. Third, the GBD 2023 dataset does not stratify melanoma by specific histological subtypes, such as separating cutaneous melanoma from acral lentiginous melanoma (ALM) or mucosal melanoma. Given that ALM is the predominant subtype in East Asian populations, the inability to analyze its specific disease burden and distinct etiological drivers independently represents a major limitation of this research. Finally, although the GBD methodology is robust, estimates for regions with developing healthcare infrastructure, such as Mongolia and Democratic People’s Republic of Korea, may be influenced by limited local diagnostic capacity and incomplete cancer-registry coverage. These limitations should be considered when interpreting and generalizing the findings of this study.”*

**Comment 1.2:** *Please provide a more detailed limitations analysis, including reliance on secondary data and exclusion of younger populations.*

**Response:** We agree with the reviewer. We have expanded the Limitations section to explicitly acknowledge that this study relies on modeled, aggregated secondary data from the GBD 2023 database rather than primary cancer-registry or patient-level data. Therefore, the findings inevitably inherit uncertainties associated with the original data sources, diagnostic practices, registry completeness, and GBD modeling procedures. We have also clarified that the analysis was intentionally restricted to adults aged 60 years and older because the study focused specifically on melanoma burden in East Asia’s aging population. Consequently, the findings should not be generalized to younger populations, and the exclusion of these populations prevents a comprehensive assessment of melanoma patterns across the entire age spectrum.

***Changes made in the manuscript (Discussion, Limitations):*** *The Limitations paragraph has been expanded as follows:“Several limitations must be acknowledged in this study. First, this study was a secondary analysis of modeled and aggregated estimates obtained from the GBD 2023 database rather than an analysis of primary cancer-registry or patient-level data. Therefore, the findings inevitably inherit uncertainties associated with the original data sources, differences in diagnostic practices and reporting quality, incomplete registry coverage, and the modeling procedures used by the GBD study. Second, our analysis was intentionally restricted to adults aged 60 years and older because the study specifically focused on the burden of malignant melanoma in East Asia’s aging population. Consequently, the findings should not be generalized to younger populations, and the exclusion of these populations prevents a comprehensive assessment of melanoma patterns across the entire age spectrum. Third, the GBD 2023 dataset does not stratify melanoma by specific histological subtypes, such as separating cutaneous melanoma from acral lentiginous melanoma (ALM) or mucosal melanoma. Given that ALM is the predominant subtype in East Asian populations, the inability to analyze its specific disease burden and distinct etiological drivers independently represents a major limitation of this research. Finally, although the GBD methodology is robust, estimates for regions with developing healthcare infrastructure, such as Mongolia and Democratic People’s Republic of Korea, may be influenced by limited local diagnostic capacity and incomplete cancer-registry coverage. These limitations should be considered when interpreting and generalizing the findings of this study.”*

**Comment 1.3:** *Please offer more detailed and actionable public health strategies, including cost-effectiveness analyses and intervention evaluations.*

**Response:** Thank you for this constructive suggestion. We agree that public health recommendations should be specific, actionable, and accompanied by an appropriate framework for evaluating their effectiveness. Accordingly, we have expanded the Discussion to prioritize targeted screening of the hands, feet, and nail beds among older adults; public education promoting routine self-examination of acral sites; strengthened dermatologic capacity in underserved regions; and improved access to modern immunotherapies. We also agree that the effectiveness and economic value of these interventions require formal evaluation. However, a cost-effectiveness analysis could not be performed in the present study

because the aggregated GBD dataset does not contain information on intervention costs, healthcare-resource utilization, screening participation, stage at diagnosis, treatment uptake, or program-level outcomes. We have therefore avoided making unsupported claims regarding

cost-effectiveness and added a recommendation that these strategies should initially be evaluated through prospective pilot programs. Such evaluations should assess screening participation and coverage, referral and diagnostic yield, stage distribution at diagnosis, treatment uptake, patient outcomes, resource requirements, and cost-effectiveness before

large-scale implementation.

**Changes made in the manuscript (Discussion):** *The public health recommendations have been expanded to include specific prevention and healthcare actions, together with a framework for future intervention and economic evaluations. The revised paragraph reads:“In light of these distinct clinical characteristics, it becomes imperative to design and implement melanoma prevention strategies that are specifically tailored to the East Asian demographic(39). Because ALM and mucosal types dominate the regional disease burden, relying primarily on Western-centric recommendations such as sun protection is unlikely to be sufficient for this specific population(8). Priority actions should include expanding early detection through targeted screening of older adults, with particular attention to the hands, feet, and nail beds(40). Public health campaigns should also promote routine self-examination of these anatomically concealed sites. Furthermore, healthcare systems should strengthen dermatologic capacity in underserved regions and improve equitable access to modern immunotherapies, thereby enabling older patients to receive timely diagnosis and appropriate treatment. These strategies should initially be implemented and assessed through prospective pilot programs. Future intervention evaluations should examine screening participation and coverage, referral and diagnostic yield, stage distribution at diagnosis, treatment uptake, patient outcomes, and healthcare-resource requirements. Formal health-economic studies should also evaluate the cost-effectiveness and feasibility of these interventions before their large-scale implementation.”*

**Comment 1.4:** *Please confirm that all data and methods are fully accessible and reproducible in accordance with PLOS ONE's data policy.*

**Response:** We confirm that the study uses publicly available, de-identified GBD 2023 data. The revised Data Availability Statement provides the direct GHDx Results Tool address. The Methods identifies the software versions, outcome definitions, joinpoint analysis, APC component, forecasting features, candidate models, chronological split, rolling validation,

model-selection metric, and uncertainty procedure needed to reproduce the analyses.

**Changes made in the manuscript (Data Availability Statement):** *The data are publicly available from the GBD 2023 database through the Global Health Data Exchange Results Tool at https://ghdx.healthdata.org/gbd-results-tool.*

**REVIEWER #2**

**Major Comments**

**Major Comment 2.1:** *The manuscript repeatedly uses "Taiwan Province of China." Please merge it with China, use "Taiwan," or remove it.*

**Response:** Thank you for this thoughtful suggestion. We carefully reviewed the geographical terminology used throughout the manuscript. Because this study directly analyzes location-specific estimates as defined and reported in the GBD 2023 Results Tool, we have retained

“Taiwan Province of China” as a separate analytical location to ensure consistency with the source database and facilitate reproducibility. Combining this data series with that of China would require additional population-weighted recalculation and would no longer directly

correspond to the estimates provided by the GBD Results Tool. To avoid possible misunderstanding, we have added a clarification in the Methods section stating that all geographical names and location classifications follow the official nomenclature of the GBD 2023 database and are used solely to maintain consistency with the source data. We have also checked the manuscript, tables, and figure legends to ensure that this terminology is used

consistently throughout. We hope this clarification adequately addresses the reviewer’s concern.

**Changes made in the manuscript (Methods, Section 2.1):** *The following clarification has been added:“East Asia was defined according to the geographical classification used in the GBD 2023 database and included China, Japan, the Republic of Korea, the Democratic People’s Republic of Korea, Mongolia, and Taiwan Province of China. All geographical names and location classifications used in this study follow the official nomenclature of the GBD 2023 Results Tool to ensure consistency with the source data and reproducibility of the analysis.”*

**Major Comment 2.2:** *Reference 10 should be updated with an appropriate and reliable source.*

**Response:** Thank you for this important suggestion. We agree that the previous reference did not adequately represent the most recent GBD data and analytical framework. We have therefore replaced Reference 10 with a recent peer-reviewed publication from the GBD 2023 Breast Cancer Collaborators, which applies the updated GBD 2023 framework to disease-burden estimation and forecasting from 1990 to 2023. Because the present study’s melanoma-specific estimates were extracted directly from the GBD 2023 Results Tool, we have also retained the direct GHDx link in the Methods and Data Availability Statement. This revision provides both a current peer-reviewed reference for the GBD 2023 analytical framework and a precise public-access route to the melanoma data analyzed in our study.

**Changes made in the manuscript (Methods, Section 2.1; Data Availability Statement):** *The previous reference has been replaced with the following updated GBD 2023 publication:“GBD 2023 Breast Cancer Collaborators. Global, regional, and national burden*

*of breast cancer among females, 1990–2023, with forecasts to 2050: a systematic analysis for the Global Burden of Disease Study 2023. Lancet Oncology. 2026;27(3):302–326. doi:10.1016/S1470-2045(25)00730-2.”*

**Major Comment 2.3:** *Please clarify whether primary data were collected to establish case identification using ICD-10 codes C43-C43.9 and Z85.82-Z85.828.*

**Response:** Thank you for requesting this clarification. No primary data were collected in this study, and the authors did not use ICD-10 codes to independently identify individual patients or adjudicate melanoma cases. All incidence, prevalence, mortality, and DALY estimates were extracted directly from the publicly available, de-identified, and aggregated GBD 2023 Results

Tool. We acknowledge that the wording in the original manuscript may have incorrectly implied that the authors performed primary case identification. We have therefore revised the Methods to clarify that malignant melanoma of the skin was defined according to the GBD

2023 cause classification, corresponding to ICD-10 category C43 (C43.0–C43.9). We also removed the previously listed codes Z85.82–Z85.828 because these ICD-10-CM codes describe a personal history of malignant skin neoplasms rather than active melanoma cases; moreover, this range includes a history of Merkel cell carcinoma and other malignant skin

neoplasms. These codes were therefore not appropriate for defining the active melanoma

estimates analyzed in this study.

**Changes made in the manuscript (Methods, Section 2.1):** *The original statement has been replaced with the following:“In this study, malignant melanoma of the skin was defined according to the GBD 2023 cause classification, corresponding to ICD-10 category C43 (C43.0–C43.9). No primary data were collected, and the authors did not independently identify or adjudicate individual cases.”The previously listed Z85.82–Z85.828 codes have been removed from the manuscript because they represent personal-history categories rather than active malignant melanoma.*

**Major Comment 2.4:** *Please clarify whether a formal IRB exemption was obtained and provide the exemption number if applicable.*

**Response:** Thank you for requesting this clarification. This study was based exclusively on

publicly available, de-identified, and aggregated data obtained from the GBD 2023 database. We did not collect primary data, access individual-level records, recruit or contact human participants, or obtain any identifiable personal information. Under the applicable institutional requirements, research based solely on publicly available and fully aggregated data does not require submission for ethics committee or institutional review board approval. Therefore, no formal IRB application was submitted, and no exemption or approval number was issued. We

have revised the manuscript to state that ethics approval and informed consent were not

required, rather than stating that the study received a formal IRB exemption.

**Changes made in the manuscript (Ethics statement):** *The original ethics statement has been replaced with the following:“This study used exclusively publicly available, de-identified, and aggregated data obtained from the Global Burden of Disease 2023 database. No individual-level records were accessed, and no human participants were recruited, contacted, or directly involved in the study. Therefore, ethics committee or institutional review board*

*approval and individual informed consent were not required. Accordingly, no ethics approval or exemption number was issued.”*

**Major Comment 2.5:** *Please add the methods and thresholds used to classify ASIR, ASPR, ASMR, and ASDR as highest, moderate, or lowest.*

**Response:** Thank you for identifying this ambiguity. We agree that the terms “highest,” “moderate,” and “lowest” could be interpreted as predefined statistical or clinical categories, although no classification thresholds were applied in this study. Rather than introducing arbitrary post hoc thresholds, we removed the original map and replaced it with Table 3, which presents the exact ASIR, ASPR, ASMR, and ASDR estimates and their corresponding 95% uncertainty intervals for each of the six included locations. The table also provides values for both sexes combined, males, and females. The revised Results now report direct numerical comparisons without assigning locations to categorical levels. Therefore, no additional classification method or threshold was required.

**Changes made in the manuscript (Results, Section 3.2; Table 3):** *The original Figure 1 and all references to its map-based categories were removed. A new Table 3 was added to report the exact values of ASIR, ASPR, ASMR, and ASDR, with 95% uncertainty intervals, for China, Taiwan (Province of China), Japan, the Democratic People’s Republic of Korea, the Republic of Korea, and Mongolia, stratified by sex. The expressions “highest,” “moderate,” and “lowest,” together with the unsupported references to inland Chinese provinces and differences between developed, coastal, and less-developed inland areas, were deleted. Section 3.2 was revised as follows:*

*“In 2023, the four age-standardized indicators of MM among the elderly population varied across the six included East Asian locations (Table 3). For both sexes combined, Taiwan (Province of China) had the highest ASIR at 2.79 per 100,000 population (95% UI: 2.02–3.68), followed by the Republic of Korea at 2.56 (95% UI: 1.64–4.29) and Japan at 2.14 (95% UI: 1.53–2.86). The corresponding ASIRs were lower in the Democratic People’s Republic of Korea at 1.25 (95% UI: 0.74–1.99), the People’s Republic of China at 0.79 (95% UI: 0.54–1.25), and Mongolia at 0.72 (95% UI: 0.47–1.19). A similar location ordering was observed for ASPR. Taiwan (Province of China) had the highest ASPR at 22.02 per 100,000 population (95% UI: 15.45–29.73), followed by the Republic of Korea at 20.39 (95% UI: 12.37–35.61) and Japan at 17.85 (95% UI: 12.60–23.82). Lower ASPR point estimates were observed in the Democratic People’s Republic of Korea at 7.43 (95% UI: 4.20–12.34), the People’s Republic of China at 5.03 (95% UI: 3.17–8.48), and Mongolia at 3.54 (95% UI: 1.95–6.54). Female ASIR and ASPR point estimates were higher than the corresponding male estimates in all six locations.*

*The location pattern of fatal outcomes differed from that of incidence and prevalence. Taiwan (Province of China) and the Democratic People’s Republic of Korea had the highest ASMR point estimates, both at 0.47 per 100,000 population, with 95% UIs of 0.41–0.55 and 0.29–0.69, respectively. They were followed by Mongolia at 0.37 (95% UI: 0.24–0.57), the Republic of Korea at 0.34 (95% UI: 0.24–0.50), the People’s Republic of China at 0.27 (95% UI: 0.19–0.39), and Japan at 0.25 (95% UI: 0.21–0.29). Taiwan (Province of China) also had the highest ASDR at 14.19 per 100,000 population (95% UI: 12.31–16.57), followed by the Democratic People’s Republic of Korea at 13.88 (95% UI: 8.72–20.26), the Republic of Korea at 9.75 (95% UI: 6.95–15.19), Mongolia at 9.55 (95% UI: 6.43–14.62), the People’s Republic of China at 7.57 (95% UI: 5.42–11.14), and Japan at 7.52 (95% UI: 6.47–8.91). Male ASDR point estimates were higher than the corresponding female estimates in all six locations. Male ASMR point estimates were higher in the People’s Republic of China, Taiwan (Province of China), Japan, and the Republic of Korea, whereas female ASMR point estimates were slightly higher in the Democratic People’s Republic of Korea and Mongolia. The complete sex-specific estimates and their corresponding 95% UIs are presented in Table 3.”*

**Major Comment 2.6:** *The Results describing Figures 2 and 3 contain several inaccuracies and misinterpretations.*

**Response:** Thank you for identifying these inaccuracies. We agree and have thoroughly rechecked the Results against the relevant figures. The reviewer refers to Figures 2 and 3 in the original submission, which have been renumbered as Figures 1 and 2, respectively, in the revised manuscript following the removal of the original Figure 1. The previous description conflated absolute numbers with age-specific rates and incorrectly characterized the distributions as uniformly unimodal. In the revised manuscript, Figure 1 is explicitly described in terms of absolute numbers, which show non-monotonic patterns with peak ages varying by sex and outcome. Figure 2 is described separately in terms of age-specific rates, which generally increase with age, although modest fluctuations occur in several age groups. This revision avoids treating absolute numbers as rates and accurately reports the sex- and indicator-specific patterns shown in the figures.

**Changes made in the manuscript (Results, Section 3.3):** *The paragraph corresponding to revised Figure 1 now reports the absolute numbers of incident cases, prevalent cases, deaths, and DALYs, including their sex-specific peak ages and values, and explicitly states that the distributions do not follow a uniform unimodal pattern. The paragraph corresponding to revised Figure 2 separately describes the age-specific incidence, prevalence, mortality, and DALY rates.*

**Major Comment 2.7:** *The statement that mortality increased after age 60 but peaked at age 55-59 is logically contradictory.*

**Response:** We agree. This contradiction resulted from conflating absolute death counts with age-specific mortality rates. In Figure 2, the combined number of deaths was greatest at ages

55–59, although the values at ages 70–74 and 75–79 were very similar. Sex-specific peaks also differed: female deaths were greatest at ages 75–79, whereas male deaths were greatest at ages

55–59. In contrast, Figure 2 shows that the age-specific mortality rate generally increased with age, despite modest fluctuations, and reached its highest value in the 95+ age group. These two measures are now described separately.

**Changes made in the manuscript (Results, Section 3.3):** *The contradictory statement was removed. The revised text reports the age- and sex-specific peaks in absolute death counts from Figure 1 separately from the mortality-rate pattern shown in Figure 2.*

**Major Comment 2.8:** *The statement that incidence reached its maximum at age 75-79 is inconsistent with the figures.*

**Response:** Thank you for pointing this out. We corrected the statement by distinguishing absolute incident counts from age-specific incidence rates. In Figure 1, the combined and male

incident counts were greatest at ages 55–59, reaching 3,071 and 1,731 cases, respectively, whereas the female incident count was greatest at ages 70–74, reaching 1,525 cases. In Figure 2, the incidence rate generally increased with age and reached its highest value in the 95+

group. The revised manuscript therefore no longer states that incidence generally peaks at ages 75–79.

**Changes made in the manuscript (Results, Section 3.3):** *The revised Results separately report the peak ages of absolute incident counts by sex and state that the age-specific incidence rate reaches its highest value in the 95+ group.*

**Major Comment 2.9:** *The statement that prevalence peaks around age 75-79 for both sexes is inconsistent with the figures.*

**Response:** We agree and have corrected the overgeneralization. The previous wording did not adequately distinguish absolute prevalent counts from age-specific prevalence rates. In Figure

1, the combined and male prevalent counts were greatest at ages 55–59, reaching 22,218 and 11,892 cases, respectively. Among females, the prevalent count was greatest at ages 70–74, although the values at ages 70–74 and 55–59 were nearly identical (10,327 and 10,326 cases, respectively). Figure 2 shows a different pattern for the prevalence rate, which increased until approximately ages 75–79 and subsequently declined in the oldest age groups. These patterns are now reported separately.

**Changes made in the manuscript (Results, Section 3.3; Figure legends):** *The revised Results report the sex-specific prevalence-count peaks shown in Figure 1 separately from the*

*age-specific prevalence-rate trajectory shown in Figure 2. The figure legends also clearly identify Figure 1 as showing absolute numbers and Figure 2 as showing age-specific rates.*

**Major Comment 2.10:** *The manuscript claims male incidence rates exceeded female rates and refers to widening beyond age 60 although Figure 4 uses calendar year and shows higher female rates.*

**Response:** We agree and have corrected this interpretation. Figure 4 presents temporal trends

by calendar year rather than patterns across age groups. The revised Results now state that female ASIR and ASPR were consistently higher than the corresponding male rates throughout 1990–2023. The previous statement that male incidence rates were higher and that the difference widened beyond age 60 has been removed. The age-specific analysis in Figure 4 is

now discussed separately and indicates that females generally had higher incidence and

prevalence rates across most age groups.

**Changes made in the manuscript (Results, Section 3.3):** *The temporal-trend paragraph now states that female ASIR and ASPR remained higher than male rates throughout the study period, with broadly stable relative sex differences. All age-based interpretations were removed from the description of Figure 4.*

**Major Comment 2.11:** *An increase in age-standardized prevalence cannot be attributed to population aging.*

**Response:** We fully agree. Because ASPR is age-standardized, changes in this measure cannot

be directly attributed to shifts in the population age structure. The revised Results describe the observed ASPR trend without assigning it to population aging. In the Discussion, population aging is considered only as a potential contributor to increases in absolute case numbers and burden, particularly when these increases occur alongside much smaller or fluctuating changes in the corresponding age-standardized rates.

**Changes made in the manuscript (Results and Discussion):** *The attribution of increasing ASPR to population aging was removed. The revised Discussion limits demographic interpretations to changes in absolute burden and clearly distinguishes these changes from trends in age-standardized rates.*

**Major Comment 2.12:** *ASMR and ASDR appear relatively flat or only slightly upward, whereas the text claims a marked increase.*

**Response:** We agree with the reviewer’s interpretation. The previous wording overstated the increases in ASMR and ASDR. The revised description of Figure 4 states that these rates

fluctuated over time rather than showing sustained or marked increases. Male ASMR and ASDR remained higher than the corresponding female rates, but the magnitude of the difference varied and did not widen consistently. This interpretation is also consistent with the Joinpoint results, which indicated only mild, statistically non-significant overall increases in ASMR and ASDR.

**Changes made in the manuscript (Results, Sections 3.3 and 3.5):** *The description of marked or sustained increases was removed. The temporal-trend paragraph now characterizes*

*ASMR and ASDR as fluctuating, while the Joinpoint analysis reports mild non-significant overall increases (AAPC = 0.23%, with the 95% confidence intervals including zero).*

**Major Comment 2.13:** *The incidence pattern dips at age 90-94 but rises again at age 95+.*

**Response:** Thank you for this careful observation. We agree and have revised the description to reflect the non-monotonic change at the oldest ages. The previous wording that incidence increased persistently with age was too absolute. The revised text states that the incidence rate

generally increased with age, showed a slight dip at ages 90–94, and subsequently rebounded to its highest level in the 95+ group.

**Changes made in the manuscript (Results, age-specific incidence analysis):** *The word “persistently” was replaced with “generally,” and the dip at ages 90–94 followed by the rebound in the 95+ group was explicitly added. The corrected sentence reads: “The incidence rate generally increased with age, showed a slight dip at ages 90–94, and rebounded to its highest level in the 95+ group.”*

**Major Comment 2.14:** *Figure 5 presents rates, so it cannot support the statement that China had the largest absolute number of deaths.*

**Response:** We agree. Figure 5 presents age-specific rates and therefore cannot be used to compare absolute numbers of deaths or DALYs among locations. We have removed the

statement concerning China’s absolute number of deaths from the description of Figure 5. Absolute numbers are now reported and compared only using Tables 1 and 2, whereas Figure 5 is used exclusively to describe age- and location-specific rate patterns.

**Changes made in the manuscript (Results, Sections 3.1 and 3.4):** *Absolute numbers and age-specific rates are now described separately and linked only to the appropriate tables or figure. China’s contribution to absolute deaths and DALYs is discussed with reference to Tables 1 and 2 rather than Figure 5.*

**Major Comment 2.15:** *Please explain how an East Asian regional average could be higher than all constituent-country rates.*

**Response:** Thank you for identifying this error. We acknowledge that the Eastern Asia series in the previous version was calculated incorrectly by summing the age-specific rates of the six included locations. Because rates should not be added directly, this calculation could produce a value higher than every constituent location. We have corrected this error. In the revised Figure 5, the black reference line represents the unweighted arithmetic mean of the six location-specific rates, calculated separately for each age group and epidemiological indicator. We used an arithmetic mean because the age- and location-specific population weights required to reconstruct a population-weighted East Asian aggregate were not available in the dataset extracted for this analysis. To avoid further misunderstanding, the line is now labeled “Arithmetic Mean” rather than “Eastern Asia” or “regional average.”

**Changes made in the manuscript (Results, Section 3.4; Figure 5):** *The incorrectly summed Eastern Asia series was removed and replaced with the unweighted arithmetic mean of the six included locations. The figure legend now labels the black line as “Arithmetic Mean.” The Results clarify that this line is calculated separately for each age group and indicator and does not represent a population-weighted or official GBD estimate for Eastern Asia. Location-specific patterns are described directly according to the individual curves displayed in Figure 5.*

**Major Comment 2.16:** *The ASDR series is volatile and includes periods of decline; it should not be described simply as upward.*

**Response:** We agree. The previous description oversimplified the ASDR trend. The revised Results now characterize ASDR as showing a small, statistically non-significant overall change from 1990 to 2023 (AAPC = 0.23%; 95% CI: −0.24%–0.71%; p = 0.336), rather than

a sustained upward trend. The segmented analysis showed a significant increase during 1990–2000, a significant decline during 2000–2009, non-significant fluctuations during the intermediate periods, and a significant increase during 2020–2023.

**Corrected text (Results, Section 3.5):** *The description of a simple or sustained upward ASDR trend was removed. ASDR is now reported as increasing significantly during 1990–2000, declining significantly during 2000–2009, fluctuating during the intermediate periods, and rebounding significantly during 2020–2023.*

**Major Comment 2.17:** *The stated 2010-2018 DALY APC segment does not exist and the reported APC values do not match Figure 6.*

**Response:** We agree and appreciate the reviewer’s careful examination. The previously reported 2010–2018 segment was incorrect and has been removed. We rechecked all segment

boundaries and APC estimates against the final Joinpoint output and regenerated Figure 6D using the same output. For the combined-sex ASDR, the final segments were 1990–2000 (APC = 1.25%; 95% CI: 0.94%–1.56%; p < 0.001), 2000–2009 (APC = −0.75%; 95% CI: −1.12% to −0.38%; p < 0.001), 2009–2012 (APC = 2.14%; 95% CI: −1.17%–5.56%; p = 0.193), 2012–

2015 (APC = −2.67%; 95% CI: −5.88%–0.65%; p = 0.107), 2015–2020 (APC = −0.55%; 95% CI: −1.66%–0.57%; p = 0.315), and 2020–2023 (APC = 2.22%; 95% CI: 0.36%–4.12%; p = 0.022).

**Changes made in the manuscript (Results, Section 3.5; Figure 6D):** *The nonexistent 2010–2018 segment and the incorrect APC values were removed. The Results and Figure 6D were updated using the same final Joinpoint output so that all segment boundaries, APC estimates, confidence intervals, and significance indicators are consistent.*

**Major Comment 2.18:** *The claim that burden was consistently higher in men conflicts with higher female incidence and prevalence in Figure 6.*

**Response:** We agree. The previous statement used the general term “burden” without specifying the outcome and was therefore misleading. The revised Results distinguish morbidity indicators from fatal outcomes. Female incidence and prevalence rates remained higher than the corresponding male rates, and both sexes showed significant long-term increases in these indicators. In contrast, mortality and DALY rates remained higher in males. Moreover, the long-term increases in ASMR and ASDR were statistically significant among

males but not among females. We have removed the generalized statement that the overall burden was consistently higher in men.

**Changes made in the manuscript (Results, Sections 3.3 and 3.5):** *The Results now state that incidence and prevalence rates were higher in females, whereas mortality and DALY rates were higher in males. The term “burden” is no longer used without identifying the specific outcome being compared.*

**Major Comment 2.19:** *Figures 7G and 7H show mortality and DALY period effects declining from 1990, not only after 2005.*

**Response:** We agree and thank the reviewer for identifying this inaccurate description. After rechecking the age–period–cohort results, we confirmed that the period rate ratios for mortality and DALYs declined throughout the entire period displayed in Figures 7G and 7H, rather than only after 2005. The corresponding Results text has been corrected.

**Corrected text (Results, Section 3.6):** *The revised text now states: “In contrast, the period rate ratios for mortality and DALYs declined continuously over the entire study period (Figure 7G, H).”*

**Major Comment 2.20:** *The projection text conflicts with Figure 8 and should describe each graph more specifically.*

**Response:** Thank you for identifying this issue. We comprehensively rechecked Figure 8 against the final prediction files and rewrote the projection section accordingly. The revised text clarifies that observations through 2023 were used to generate forecasts for 2024–2040. For each location–indicator series, the estimates reported in the Results were obtained from

the model selected using rolling time-series cross-validation, and the corresponding 95% prediction intervals were generated using the residual-bootstrap procedure. ASIR, ASPR, ASMR, and ASDR are now described separately by location. The revised text characterizes ASIR and ASPR as remaining broadly stable with modest location-specific increases or decreases. Similarly, ASMR and ASDR are described as remaining broadly stable or changing

modestly, rather than uniformly declining or entering an identical plateau across all locations. Exact 2040 point forecasts and 95% prediction intervals are provided for all six locations, and deterministic interpretations of the projected values have been avoided.

**Changes made in the manuscript (Results, Section 3.7; Figure 8):** *The projection section has been divided into separate subsections for ASIR/ASPR and ASMR/ASDR. Each subsection reports the location-specific 2040 point forecasts with residual-bootstrap 95% prediction intervals and describes the heterogeneous trajectories displayed in Figure 8. The forecast period has been corrected to 2024–2040, and statements implying uniform declines, uniform plateaus, or deterministic future outcomes have been removed.*

**Major Comment 2.21:** *A dedicated limitations section is required because the study relies on secondary modeled data.*

**Response:** We agree and have added a dedicated limitations paragraph to the Discussion. The revised paragraph explicitly states that this study is a secondary analysis of publicly available, de-identified, aggregated, and modeled GBD 2023 estimates and therefore cannot replace primary cancer-registry or patient-level research. It also acknowledges that the GBD dataset does not stratify melanoma estimates by histological subtype, including cutaneous melanoma, acral lentiginous melanoma (ALM), and mucosal melanoma. This limitation is particularly relevant in East Asia, where ALM represents an important melanoma subtype. In addition, estimates for locations with more limited diagnostic capacity and cancer-registry coverage, particularly Mongolia and the Democratic People’s Republic of Korea, may be affected by

differences in case ascertainment and reporting. Because the study was intentionally restricted to older adults, its findings should not be generalized to younger populations. Finally, the

projections extrapolate historical patterns and may not capture future changes in diagnostic practices, treatment availability, healthcare policies, or other unforeseen factors. We have consequently limited causal interpretations and interpreted cross-location comparisons and future projections cautiously.

**Changes made in the manuscript (Discussion, Limitations):** *A dedicated limitations paragraph now addresses the secondary and modeled nature of the GBD data, the absence of patient-level information, the lack of histological-subtype stratification, differences in*

*diagnostic and registry coverage, the exclusion of younger populations, and the uncertainty inherent in extrapolating historical trends to 2040.*

**Minor Comments**

**Minor Comment 2.1:** *Replace "longitudinal trends" with "time trends."*

**Response:** We agree that “time trends” is more appropriate for describing repeated population-

level estimates over calendar time. We have replaced “longitudinal trends” with “time trends” in the revised manuscript.

**Changes made in the manuscript (Introduction):** *The relevant sentence now reads: “To map the time trends of MM, this research leverages the GBD 2023 data, assessing the disease burden in East Asia's elderly from 1990 to 2023 and projecting its trajectory through 2040.”*

**Minor Comment 2.2:** *Define APC and AAPC in full at first use.*

**Response:** We agree. In the revised Methods, both abbreviations are now written in full at their first use in the Joinpoint regression section. APC is defined as annual percentage change, and AAPC is defined as average annual percentage change. Because APC is also used to denote

the age–period–cohort model, its alternative meaning is separately defined at first use in the subsequent age–period–cohort analysis section.

**Changes made in the manuscript (Methods, Sections 2.3.2 and 2.3.3):** *The revised Joinpoint regression section now states: “Joinpoint regression analysis was used to identify inflection points in the time trends of MM indicators and to characterize segment-specific changes. A log-linear regression model was fitted to each segment to estimate the annual percentage change (APC), whereas the overall trend across the full study period was summarized using the average annual percentage change (AAPC) and its 95% confidence interval (CI).” In Section 2.3.3, APC is separately defined as age–period–cohort when the APC model is first introduced.*

**Minor Comment 2.3:** *Move the specified text to after Table 1 and before Table 2.*

**Response:** Thank you for this helpful suggestion. We agree that positioning the specified text between Tables 1 and 2 provides a clearer correspondence between the narrative and the tabulated results. We have therefore reinserted Tables 1 and 2 into the manuscript and moved the specified text to immediately after Table 1 and before Table 2, as recommended.

**Changes made in the manuscript (Results, Section 3.1):** *Table 1 is now placed immediately after the paragraph reporting deaths and ASMR. The paragraph describing DALYs and ASDR has been moved to follow Table 1, and Table 2 is positioned immediately after this paragraph. The revised sequence is therefore: deaths and ASMR results, Table 1, DALY and ASDR results, and Table 2.*

**Minor Comment 2.4:** *Rename the subheader so it accurately identifies the melanoma metric being discussed.*

**Response:** We agree. The previous subheader used the general term “burden,” although the corresponding section specifically describes age-specific rates and regional rate patterns. We have therefore revised the subheader to identify the metric more precisely. We have also added a separate subheader for the Joinpoint analysis so that the age-specific regional analysis and temporal analysis are clearly distinguished.

**Changes made in the manuscript (Results, Sections 3.4 and 3.5):** *Section 3.4 has been renamed “Age-specific and regional patterns of MM rates among the elderly population in East Asia,” and Section 3.5 is now titled “Joinpoint regression analysis of age-standardized MM rates.”*

**Minor Comment 2.5:** *Clarify whether the heavier male burden refers to counts or standardized rates and cite the supporting figures.*

**Response:** We agree that the previous expression “heavier male burden” was insufficiently specific. The revised Results now distinguish absolute numbers from rates and identify the epidemiological indicator being compared. Female incidence and prevalence rates were generally higher than the corresponding male rates, whereas male mortality and DALY rates

were higher than the corresponding female rates. The age-specific comparisons are supported by Figure 3, the temporal sex-specific patterns by Figure 4, and the age-standardized Joinpoint results by Figure 6. We have removed the ambiguous generalized statement that males had a heavier overall burden.

**Changes made in the manuscript (Results, Sections 3.1, 3.3 and 3.5):** *Absolute death and DALY counts are now reported separately from ASMR and ASDR. Sex differences are described using the specific indicators and are linked to Figures 3, 4, and 6, as appropriate.*

**Minor Comment 2.6:** *Remove the world map from Figure 1 and focus on East Asia or included countries.*

**Response:** Thank you for this helpful suggestion. We agree that the original world map was not sufficiently focused on the geographical scope of this study and made the location-specific results difficult to read. We have therefore removed the original Figure 1 entirely and replaced it with a table presenting the corresponding numerical estimates for the six included East Asian locations. The replacement table reports the 2023 age-standardized incidence rate (ASIR), age-standardized prevalence rate (ASPR), age-standardized mortality rate (ASMR), and age-standardized DALY rate (ASDR) per 100,000 population. For each location and indicator, estimates are presented for both sexes combined, males, and females, together with their corresponding 95% uncertainty intervals. This tabular presentation allows readers to compare the exact values across locations, indicators, and sexes more clearly than the original map.

**Changes made in the manuscript (Results, Section 3.2; original Figure 1 and replacement table):** *The four-panel world map previously presented as Figure 1, together with its original figure legend, has been deleted. It has been replaced with a table entitled “**Age-standardized rates of malignant melanoma among the older population in six East Asian locations in 2023, by sex.” The rows of the new table list the People’s Republic of China, Taiwan (Province of China), Japan, the Democratic People’s Republic of Korea, the Republic of Korea, and Mongolia. The columns are organized into four indicator blocks ASIR, ASPR, ASMR, and ASDR and each block contains estimates for both sexes combined, males, and females. All rates are reported per 100,000 population as point estimates with 95% uncertainty intervals. References to “Figure 1A–D” in Section 3.2 have been replaced with references to the new table, and descriptions based on map colors or spatial categories have been removed.*

**Minor Comment 2.7:** *Figures 2 and 3 have identical titles and need distinct, specific captions.*

**Response:** We agree. To distinguish the two figures while maintaining consistency with the expressions used within the figures, we revised the captions to specify that Figure 1 presents the absolute numbers of MM-related outcomes, whereas Figure 2 presents the corresponding

age-specific rates.

**Changes made in the manuscript (Figure legends):**

*“Figure 1. Age and sex differences in the absolute numbers of MM-related outcomes among the elderly population in East Asia in 2023. (A) Incident cases. (B) Prevalent cases. (C) Deaths. (D) DALYs.”*

*“Figure 2. Age and sex differences in age-specific rates of MM among the elderly population in East Asia in 2023. (A) Incidence rates. (B) Prevalence rates. (C) Death rates. (D) DALY rates.”*

**Minor Comment 2.8:** *Label each panel of Figure 7 with its corresponding measure.*

**Response:** We agree. We have retained the original title and terminology of Figure 7 while revising the legend to identify the measure corresponding to each panel individually. Panels A–D now separately identify the longitudinal age curves, panels E–H identify the period

relative risks, and panels I–L identify the cohort relative risks for incidence, prevalence, deaths, and DALYs.

**Changes made in the manuscript (Figure 7 legend): The revised legend reads:**

*“Figure 7. Age–period–cohort analysis results for MM burden among the elderly population across East Asian countries, 1990–2023. (A) Longitudinal age curve for incidence. (B) Longitudinal age curve for prevalence. (C) Longitudinal age curve for deaths. (D) Longitudinal age curve for DALYs. (E) Period RR for incidence. (F) Period RR for prevalence.*

*(G) Period RR for deaths. (H) Period RR for DALYs. (I) Cohort RR for incidence. (J) Cohort RR for prevalence. (K) Cohort RR for deaths. (L) Cohort RR for DALYs.”*

**Minor Comment 2.9:** *Figure 8 is blurred and too small to read.*

**Response:** We agree and thank the reviewer for pointing out this presentation issue. We have regenerated Figure 8 as a standalone high-resolution image and increased the panel dimensions and the sizes of the axis labels, legends, and other text elements to improve readability. The

revised high-resolution figure will be uploaded separately with the revised submission in accordance with the journal’s figure requirements. We have retained the original structure of the figure and made minor revisions to the legend to clarify the outcomes presented in each group of panels.

**Changes made in the manuscript (Figure 8 and its legend): The revised legend reads:**

“Figure 8 Future projections to 2040 for MM in the People’s Republic of China, Japan, the Republic of Korea, the Democratic People’s Republic of Korea, Mongolia, and Taiwan (Province of China).(A1-A6) Incidence; (B1-B6) Prevalence; (C1-C6) Mortality; (D1-D6) DALYs.”

**REVIEWER #3**

**Statistical and Presentation Comments**

**Comment 3.1:** *The linear model y = beta0 + beta1 age + beta2 period + beta3 cohort is non-identifiable because cohort = period - age. Please consider an identifiable APC method such as those implemented in the Epi package.*

**Response:** Thank you for identifying this important statistical issue. We agree that the unrestricted linear effects of age, period, and birth cohort cannot be uniquely estimated because birth cohort is exactly determined by calendar period and age. We also appreciate the reviewer’s recommendation regarding the Epi package. The equation presented in the previous version did not accurately describe the analytical framework used and could incorrectly imply that three independent coefficients had been identified. We have therefore removed this equation and the corresponding interpretation of separate (\beta) coefficients. The revised Methods now clarifies that the APC analysis was based on the estimable-functions framework implemented in the National Cancer Institute APC Web Tool. This framework addresses the identification problem by reporting identifiable APC functions, including longitudinal age curves, period rate ratios, and cohort rate ratios, rather than attempting to estimate three unrestricted linear coefficients independently. Accordingly, the APC findings are interpreted descriptively as age-, period-, and cohort-related patterns and not as independent causal effects. No separate age, period, or cohort regression coefficients are reported or interpreted in the revised manuscript.

**Changes made in the manuscript (Methods, Section 2.3.3; Results, Section 3.6; Discussion):** *In Section 2.3.3, we deleted the non-identifiable equation (*$Y_{t}=\beta_{0}+\beta_{1}\left( {Age}_{t} \right)+\beta_{2}\left( {Period}_{t} \right)+\beta_{3}\left( {Cohort}_{t} \right)+\epsilon_{t}$*). We also deleted the statements that the model “disentangled the independent contributions” of age, period, and cohort and*  $\beta_{1}$*,* $\beta_{2}$*, and* $\beta_{3}$ *represented their separate contributions. These passages were replaced with the following description:*

*“Age–period–cohort (APC) analysis was performed using the estimable-functions framework implemented in the National Cancer Institute APC Web Tool. Because birth cohort is exactly determined by calendar period and age (cohort = period − age), the unrestricted linear effects of age, period, and cohort cannot be estimated independently. Accordingly, individual regression coefficients were not interpreted as uniquely identifiable or causal effects. Instead, the analysis focused on identifiable APC functions, including longitudinal age curves, period rate ratios (RRs), and cohort RRs. The APC results were interpreted descriptively and were not used to establish independent causal effects of age, period, or birth cohort.”*

*We also added the methodological reference by Rosenberg et al. for the NCI APC Web Tool. In Section 3.6, the APC results are now reported exclusively as longitudinal age curves and period and cohort RR patterns. Wording implying that the analysis established independent age, period, or cohort effects has been removed. In the Discussion, causal interpretations of the APC findings have been replaced with cautious descriptions of age-related, temporal, and generational patterns.*

**Comment 3.2:** *Interpretive discussion is mixed into the Results and should be moved to the Discussion.*

**Response:** We agree. We carefully reviewed the entire Results section and revised it to focus exclusively on the quantitative findings displayed in the tables and figures, including absolute numbers, rates, uncertainty intervals, Joinpoint estimates, age–period–cohort patterns, and

forecast values. Explanations concerning histological subtypes, diagnostic access, delayed

detection, cancer-registry coverage, healthcare infrastructure, treatment advances, behavioral factors, and potential mechanisms underlying the observed patterns have been consolidated in the Discussion. We have also removed causal or explanatory wording from the Results where the analyses support only descriptive associations.

**Changes made in the manuscript (Results, Sections 3.1–3.7; Discussion):** *Sections 3.1–3.7 were revised to present the numerical and graphical findings without clinical or causal*

*interpretation. Explanatory material concerning ALM, diagnostic practices, healthcare access, treatment availability, population aging, and other potential determinants was moved to the Discussion. Interpretive statements concerning the limitations of the forecasts were also consolidated in the Discussion and Limitations section.*

**Comment 3.3:** *EPAC/EAPC, AAPC, and APC are not clearly explained or distinguished.*

**Response:** Thank you for highlighting the need to distinguish these related trend measures more clearly. We understand “EPAC” in the comment to refer to EAPC. In the revised manuscript, we have clarified that the estimated annual percentage change (EAPC) is obtained from a single log-linear regression fitted over the entire study period and is used to summarize

the overall annual trend in an age-standardized rate. In contrast, the annual percentage change (APC) is estimated separately for each time segment identified by Joinpoint regression and describes the annual trend within that specific segment. The average annual percentage change (AAPC) provides a time-weighted summary of the segment-specific APCs over the full study period. All three measures are expressed as relative percentage changes per year rather than as additive changes in the original rate units. Because APC is also used as the abbreviation for the age–period–cohort model, the latter is written in full at first use and is clearly distinguished by its analytical context.

**Changes made in the manuscript (Methods, Sections 2.3.1–2.3.3):** *The revised Methods now define EAPC as the overall annual percentage trend estimated using a single log-linear regression across the full study period, APC as the segment-specific annual*

*percentage trend estimated by Joinpoint regression, and AAPC as the time-weighted summary of the Joinpoint APCs across the full period. The age–period–cohort model is separately defined in full when first introduced.*

**Comment 3.4:** *Please clarify whether APC/EAPC is on an additive or multiplicative scale and how values such as EAPC = 50.70 should be interpreted.*

**Response:** Thank you for requesting this clarification. We understand “EPAC” in the comment

to refer to EAPC. In this study, EAPC is expressed as an estimated annual relative percentage change in the corresponding age-standardized rate, rather than as an additive change in the original rate units.

Specifically, the EAPC value of 50.79 reported for the ASMR of Eastern Asia in Table 1 is a model-based point estimate indicating an estimated average annual relative change of 50.79% over the study period. It does not mean that the ASMR increased by 50.79 units per 100,000 population, nor does it represent the total percentage change between 1990 and 2023. However, this point estimate has a very wide 95% confidence interval that includes 0. Therefore, the estimated trend is not statistically significant, and the positive point estimate should not be interpreted in isolation as evidence that the ASMR truly increased by 50.79% per year. The wide confidence interval indicates substantial uncertainty regarding both the direction and magnitude of the estimated trend. Accordingly, our interpretation is based on the point estimate together with its 95% confidence interval, and the change in Eastern Asia’s ASMR is described cautiously rather than as a significant increase.

The same principle applies to APC and AAPC: APC describes the relative annual percentage change within a specific Joinpoint segment, whereas AAPC summarizes the relative annual percentage change across the full study period. For all three measures, statistical significance is determined by whether the corresponding 95% confidence interval excludes 0.

**Changes made in the manuscript (Methods, Sections 2.3.1–2.3.2; Results, Section 3.1; Table 1 footnote):** The revised manuscript now clarifies that EAPC, APC, and AAPC are relative percentage changes rather than absolute changes in rates per 100,000 population. The interpretation of the Eastern Asia ASMR estimate has been clarified to indicate that, although the EAPC point estimate was positive, its 95% confidence interval included 0 and therefore did not indicate a statistically significant trend. A corresponding explanatory note has also been added to Table 1.

**Comment 3.5:** *Because sex disparities are emphasized, please consider breaking location values down further by sex and possibly age.*

**Response:** Thank you for this valuable suggestion. We have expanded the presentation of location-specific results by sex and age. The original map has been removed and replaced with Table 3, which reports ASIR, ASPR, ASMR, and ASDR for each of the six included East Asian locations. For every location and indicator, the table provides estimates for both sexes combined, males, and females, together with their corresponding 95% uncertainty intervals. This revision allows direct comparison of sex-specific differences within each location. In addition, Figure 5 presents age-specific incidence, prevalence, mortality, and DALY rates across the same six locations, thereby showing how regional differences vary across age groups. We did not combine location, sex, age, and all four indicators into a single three-way table because this would produce an excessively large and difficult-to-read presentation. Instead, Table 3 provides the location-by-sex comparisons, while Figure 5 provides the complementary location-by-age comparisons.

**Changes made in the manuscript (Results, Sections 3.2 and 3.4; Table 3; Figure 5)**: First, the original world-map-based Figure 1 was deleted and replaced with Table 3. The rows of Table 3 list the People’s Republic of China, Taiwan (Province of China), Japan, the Democratic People’s Republic of Korea, the Republic of Korea, and Mongolia. The columns are divided into four indicator blocks ASIR, ASPR, ASMR, and ASDR and each block contains separate estimates for both sexes combined, males, and females. All values are reported per 100,000 population as point estimates with 95% uncertainty intervals. Second, Section 3.2 was rewritten to report the location-specific and sex-specific differences shown in Table 3, including the observation that female ASIR and ASPR point estimates were higher than the corresponding male estimates in all six locations, whereas male ASDR point estimates were higher than the corresponding female estimates. Third, Section 3.4 and Figure 5 present incidence, prevalence, mortality, and DALY rates by age group for each of the six locations, allowing location-specific patterns to be compared across age groups.

**Comment 3.6:** *Figure 1 is difficult to read and should be rescaled to the target locations.*

**Response:** Thank you for this suggestion. We agree that the original world map was difficult to read and was not sufficiently focused on the six locations included in this study. Rather than simply rescaling the original map, we removed Figure 1 entirely and replaced it with a numerical table. The replacement table presents the exact ASIR, ASPR, ASMR, and ASDR estimates for each of the six included East Asian locations and therefore provides a clearer and more informative comparison than the original map. It also includes sex-specific estimates and 95% uncertainty intervals, which could not be displayed clearly in the map.

**Changes made in the manuscript (Results, Section 3.2; original Figure 1; Table 3):** The original four-panel world map and its figure legend were deleted. A new Table 3 was inserted to present the 2023 ASIR, ASPR, ASMR, and ASDR for the People’s Republic of China, Taiwan (Province of China), Japan, the Democratic People’s Republic of Korea, the Republic of Korea, and Mongolia. For each indicator, Table 3 contains separate columns for both sexes combined, males, and females, and reports all estimates per 100,000 population with their corresponding 95% uncertainty intervals. References to “Figure 1A–D” in Section 3.2 were replaced with references to Table 3, and the previous descriptions based on map colors and geographical categories were replaced with direct numerical comparisons of the six locations.

**Comment 3.7:** *Figure 2 uses unclear axis wording such as "numbers of incidence"; please clarify whether it means incident or prevalent cases.*

**Response:** Thank you for identifying this unclear wording. The expression “numbers of incidence” in the original figure was intended to represent the absolute number of incident cases, not the incidence rate. Similarly, “numbers of prevalence” referred to the absolute number of prevalent cases. We agree that these expressions were grammatically imprecise and could be misunderstood. We have therefore revised the axis labels and figure legend to use standard epidemiological terminology. Figure 1 now presents absolute numbers of incident cases, prevalent cases, deaths, and DALYs, whereas Figure 2 presents the corresponding age-specific rates.

**Changes made in the manuscript (Results, Section 3.3; Figure 2 and its legend):** *In Figure 1A, the x-axis label “The Numbers of Incidence” was replaced with “Number of incident cases.” In Figure 1B, “The Numbers of Prevalence” was replaced with “Number of prevalent cases.” The axis labels in Figure 1C and Figure 1D were standardized as “Number of deaths” and “Total DALYs,” respectively. The figure legend was revised to read: “**Figure 1. Age and sex differences in the absolute numbers of MM-related outcomes among the elderly population in East Asia in 2023. (A) Incident cases. (B) Prevalent cases. (C) Deaths. (D) DALYs.” In Section 3.3, the corresponding Results text now consistently uses “incident cases,” “prevalent cases,” “deaths,” and “DALYs” and does not refer to any of these absolute quantities as rates.*

**Comment 3.8:** *A bar plot is not ideal for the numeric time trends in Figure 4; dotted or dashed lines may be clearer.*

**Response:** Thank you for this helpful visualization suggestion. We agree that the original figure was difficult to interpret because it combined annual absolute numbers and age-standardized rates in the same panels using bars, lines, uncertainty intervals, and two different y-axes. To improve clarity, we divided the original combined figure into two separate figures according to the type of epidemiological measure. The revised Figure 3 presents annual absolute numbers using grouped bars, whereas the revised Figure 4 presents ASIR, ASPR, ASMR, and ASDR exclusively as connected line series. Male and female rate series in Figure 4 are distinguished by different colors, and their 95% uncertainty intervals are displayed as shaded bands. Separating the absolute numbers from the age-standardized rates removes the previous dual-axis overlay and makes the temporal rate patterns substantially easier to follow.

**Changes made in the manuscript (Results, Section 3.3; Figures 3 and 4 and their legends):** The original combined Figure 4 was divided into two standalone figures. The new Figure 3 contains four panels showing the annual absolute numbers of incident cases, prevalent cases, deaths, and DALYs from 1990 to 2023. Male and female values are displayed as separate grouped bars, with their corresponding 95% uncertainty intervals shown as error bars. The new Figure 4 contains four panels showing ASIR, ASPR, ASMR, and ASDR over the same period. In Figure 4, female rates are displayed as orange connected lines and male rates as blue connected lines, with sex-specific 95% uncertainty intervals presented as shaded bands. The previous combination of bars and rate lines on dual y-axes was removed. Section 3.3 was also revised so that statements concerning changes in absolute numbers refer to Figure 3, whereas statements concerning age-standardized rate trends refer to Figure 4.

The revised legends read:

“Figure 3. Temporal trends in the absolute numbers of MM-related outcomes among the elderly population in East Asia, 1990–2023. (A) Incident cases. (B) Prevalent cases. (C) Deaths. (D) DALYs.”

“Figure 4. Temporal trends in age-standardized rates of MM among the elderly population in East Asia, 1990–2023. (A) ASIR. (B) ASPR. (C) ASMR. (D) ASDR.”

**Comment 3.9:** *The word "burden" is sometimes ambiguous as to whether it denotes one metric or all four epidemiologic indicators.*

**Response:** Thank you for raising this point. In this manuscript, “MM burden” is not intended to denote a separate epidemiological metric. It is used as a collective descriptive term encompassing the four outcomes examined in this study: incidence, prevalence, mortality, and DALYs. The term is retained only in general contexts, such as the title, study objective, section headings, and overall summary, where all four outcomes are being considered together. When reporting or comparing a specific finding, we identify the corresponding indicator directly—for example, incident cases or ASIR, prevalent cases or ASPR, deaths or ASMR, and DALYs or ASDR. We have reviewed the manuscript to ensure that the intended indicator is clear from the context and that “burden” is not interpreted as an additional or independent measure.

**Changes made in the manuscript (Results, Sections 3.1–3.7; table and figure legends):** *No additional definition or new epidemiological variable was introduced. The general term “MM burden” was retained only where incidence, prevalence, mortality, and DALYs are discussed collectively. In the quantitative Results, the corresponding indicators are now named directly: Section 3.1 reports deaths, ASMR, DALYs, and ASDR; Section 3.2 reports ASIR, ASPR, ASMR, and ASDR separately; Section 3.3 distinguishes incident cases, prevalent cases, deaths, and DALYs from their corresponding rates; Section 3.5 reports Joinpoint trends for ASIR, ASPR, ASMR, and ASDR; and Section 3.7 reports projections separately for each of these four standardized rates. The table and figure legends likewise identify the specific outcome displayed in each panel or column.*

**Comment 3.10:** *Please clarify whether age standardization was performed by the authors or supplied by GBD.*

**Response:** Thank you for requesting this clarification. The ASIR, ASPR, ASMR, and ASDR estimates used in this study were extracted directly from the GBD 2023 Results Tool. Age standardization was performed within the GBD framework using the GBD standard population. The authors did not construct a new standard population or independently recalculate the age-standardized rates. We used the standardized estimates supplied by GBD for cross-location and temporal comparisons and subsequently applied the trend and forecasting methods described in the manuscript.

**Changes made in the manuscript (Methods, Sections 2.1 and 2.2):** *The previous sentence“All burden indicators were age-standardized against the GBD 2023 global standard population and reported as per 100,000 rates via direct standardization”was deleted because it could incorrectly imply that direct standardization was performed by the authors. It was replaced with the following statement: “Age-standardized incidence, prevalence, mortality, and DALY rates (ASIR, ASPR, ASMR, and ASDR) were extracted directly from the GBD 2023 Results Tool. These estimates were standardized by GBD using the GBD standard population and are expressed per 100,000 population.” Section 2.1 now also identifies the GBD 2023 Results Tool as the direct source of both the absolute estimates and the GBD-provided age-standardized rates.*

**Comment 3.11:** *Age-standardized trends should not be attributed to population aging.*

**Response:** We fully agree. Age-standardized rates account for differences and changes in population age structure and therefore should not be interpreted as increasing because of population aging. We have revised the Abstract, Results, Discussion, and Conclusion to maintain a clear distinction between changes in absolute numbers and changes in age-standardized rates. Population aging and expansion of the older population are now discussed only as possible contributors to increases in the absolute numbers of deaths and DALYs. Changes in ASIR, ASPR, ASMR, and ASDR are interpreted separately and are not attributed to population aging. We have also replaced deterministic causal wording with a more cautious interpretation: the divergence between rapidly increasing absolute numbers and comparatively small changes in ASMR and ASDR is consistent with, but does not independently prove, an important contribution from demographic change.

**Changes made in the manuscript (Abstract; Results, Section 3.3; Discussion; Conclusion; Figures 3 and 4):** *In the Abstract, the statement “indicating that demographic aging primarily drives absolute growth” was deleted. It was replaced with: “The marked increases in the absolute numbers of deaths and DALYs, together with the comparatively small changes in ASMR and ASDR, are consistent with an important contribution of demographic change, including expansion of the older population, to the increase in absolute MM-related outcomes.” In the Discussion, the statement that the growing burden was “predominantly fueled by demographic aging, rather than by changes in the underlying risk of the disease” was removed and replaced with: “The marked increases in absolute deaths and DALYs, together with the comparatively small changes in ASMR and ASDR, suggest that demographic change may have contributed substantially to the increase in absolute MM-related outcomes; however, this comparison does not establish a causal effect of population aging.” Interpretations of ASIR, ASPR, ASMR, and ASDR now refer only to changes in the standardized rates themselves. In addition, the original combined temporal-trend figure was divided into Figure 3, which displays absolute numbers, and Figure 4, which displays age-standardized rates, so that the two types of measures are visually and conceptually separated. The Conclusion was revised to avoid attributing any age-standardized trend to population aging.*

We sincerely thank the Academic Editor and Reviewers for their thoughtful and constructive comments, which have substantially improved the accuracy, methodological clarity, and overall quality of our manuscript. We hope that the revisions and point-by-point responses have adequately addressed all concerns. We greatly appreciate your time and consideration and look forward to your further evaluation of our revised manuscript.

Sincerely,

Yongfeng Wang, MD

Lanzhou University

wangyf1229@126.com

2026.08.05
