# Supplementary material for: Burden of malignant melanoma among the elderly population in East Asia from 1990 to 2023 and machine learning projection to 2040
Source: PLoS One. 2026 Sep 11;21(9):e0357767. doi: 10.1371/journal.pone.0357767 (PMC13568703; doi:10.1371/journal.pone.0357767)
Supplement: Peer review history

# Peer review: Decision Letter 0

**DOI:** 10.1371/journal.pone.0357767.r001

**Authors:** Pengpeng Ye

24 Jun 2026

Dear Dr. Wang,

Thank you for submitting your manuscript to PLOS ONE. After careful consideration, we feel that it has merit but does not fully meet PLOS ONE’s publication criteria as it currently stands. Therefore, we invite you to submit a revised version of the manuscript that addresses the points raised during the review process.

Please submit your revised manuscript by Aug 07 2026 11:59PM. If you will need more time than this to complete your revisions, please reply to this message or contact the journal office at plosone@plos.org. When you're ready to submit your revision, log on to https://www.editorialmanager.com/pone/ and select the 'Submissions Needing Revision' folder to locate your manuscript file.

- A letter that responds to each point raised by the academic editor and reviewer(s). You should upload this letter as a separate file labeled 'Response to Reviewers'.
- A marked-up copy of your manuscript that highlights changes made to the original version. You should upload this as a separate file labeled 'Revised Manuscript with Track Changes'.
- An unmarked version of your revised paper without tracked changes. You should upload this as a separate file labeled 'Manuscript'.

If you would like to make changes to your financial disclosure, please include your updated statement in your cover letter. Guidelines for resubmitting your figure files are available below the reviewer comments at the end of this letter.

If applicable, we recommend that you deposit your laboratory protocols in protocols.io to enhance the reproducibility of your results. Protocols.io assigns your protocol its own identifier (DOI) so that it can be cited independently in the future. For instructions see: https://journals.plos.org/plosone/s/submission-guidelines#loc-laboratory-protocols. Additionally, PLOS ONE offers an option for publishing peer-reviewed Lab Protocol articles, which describe protocols hosted on protocols.io. Read more information on sharing protocols at https://plos.org/protocols?utm_medium=editorial-email&utm_source=authorletters&utm_campaign=protocols.

As the corresponding author, your ORCID iD is verified in the submission system and will appear in the published article. PLOS supports the use of ORCID, and we encourage all coauthors to register for an ORCID iD and use it as well. Please encourage your coauthors to verify their ORCID iD within the submission system before final acceptance, as unverified ORCID iDs will not appear in the published article. *Only* the individual author can complete the verification step; PLOS staff *cannot* verify ORCID iDs on behalf of authors.

We look forward to receiving your revised manuscript.

Kind regards,

Pengpeng Ye

Academic Editor

PLOS One

Journal Requirements:

When submitting your revision, we need you to address these additional requirements.

1. Please ensure that your manuscript meets PLOS ONE's style requirements, including those for file naming. The PLOS ONE style templates can be found at

https://journals.plos.org/plosone/s/file?id=wjVg/PLOSOne_formatting_sample_main_body.pdf and

https://journals.plos.org/plosone/s/file?id=ba62/PLOSOne_formatting_sample_title_authors_affiliations.pdf

2. We note that the grant information you provided in the ‘Funding Information’ and ‘Financial Disclosure’ sections do not match.

When you resubmit, please ensure that you provide the correct grant numbers for the awards you received for your study in the ‘Funding Information’ section.

3. Thank you for stating the following financial disclosure:

“This work was funded by the 2021 Central-Guided Local Science and Technology Development Fund (ZYYDDFFZZJ-1), GanSu Joint Scientific Research Fund Major Project under Grant (No.23JRRA1537), National Natural Science Foundation of China (No. 82360498), Gansu Da Vinci robot high-end diagnosis and treatment team construction project, and Gansu Province Excellent Doctor Fund Project (23JRRA1320).”

Please state what role the funders took in the study.  If the funders had no role, please state: "The funders had no role in study design, data collection and analysis, decision to publish, or preparation of the manuscript." If this statement is not correct you must amend it as needed.

Please include this amended Role of Funder statement in your cover letter; we will change the online submission form on your behalf.

4. We note that your Data Availability Statement is currently as follows:

“All relevant data are within the manuscript and its Supporting Information files.”

Please confirm at this time whether or not your submission contains all raw data required to replicate the results of your study. Authors must share the “minimal data set” for their submission. PLOS defines the minimal data set to consist of the data required to replicate all study findings reported in the article, as well as related metadata and methods (https://journals.plos.org/plosone/s/data-availability#loc-minimal-data-set-definition).

For example, authors should submit the following data:

- The values behind the means, standard deviations and other measures reported;

- The values used to build graphs;

- The points extracted from images for analysis.

Authors do not need to submit their entire data set if only a portion of the data was used in the reported study.

If your submission does not contain these data, please either upload them as Supporting Information files or deposit them to a stable, public repository and provide us with the relevant URLs, DOIs, or accession numbers. For a list of recommended repositories, please see https://journals.plos.org/plosone/s/recommended-repositories.

If there are ethical or legal restrictions on sharing a de-identified data set, please explain them in detail (e.g., data contain potentially sensitive information, data are owned by a third-party organization, etc.) and who has imposed them (e.g., an ethics committee). Please also provide contact information for a data access committee, ethics committee, or other institutional body to which data requests may be sent. If data are owned by a third party, please indicate how others may request data access.

5. We note that Figure 1 in your submission contain map images which may be copyrighted. All PLOS content is published under the Creative Commons Attribution License (CC BY 4.0), which means that the manuscript, images, and Supporting Information files will be freely available online, and any third party is permitted to access, download, copy, distribute, and use these materials in any way, even commercially, with proper attribution. For these reasons, we cannot publish previously copyrighted maps or satellite images created using proprietary data, such as Google software (Google Maps, Street View, and Earth). For more information, see our copyright guidelines: http://journals.plos.org/plosone/s/licenses-and-copyright.

We require you to either (1) present written permission from the copyright holder to publish these figures specifically under the CC BY 4.0 license, or (2) remove the figures from your submission:

1) You may seek permission from the original copyright holder of Figure 1 to publish the content specifically under the CC BY 4.0 license.

We recommend that you contact the original copyright holder with the Content Permission Form (http://journals.plos.org/plosone/s/file?id=7c09/content-permission-form.pdf) and the following text:

“I request permission for the open-access journal PLOS ONE to publish XXX under the Creative Commons Attribution License (CCAL) CC BY 4.0 (http://creativecommons.org/licenses/by/4.0/). Please be aware that this license allows unrestricted use and distribution, even commercially, by third parties. Please reply and provide explicit written permission to publish XXX under a CC BY license and complete the attached form.”

Please upload the completed Content Permission Form or other proof of granted permissions as an ""Other"" file with your submission.

In the figure caption of the copyrighted figure, please include the following text: “Reprinted from [ref] under a CC BY license, with permission from [name of publisher], original copyright [original copyright year].”

2) If you are unable to obtain permission from the original copyright holder to publish these figures under the CC BY 4.0 license or if the copyright holder’s requirements are incompatible with the CC BY 4.0 license, please either i) remove the figure or ii) supply a replacement figure that complies with the CC BY 4.0 license. Please check copyright information on all replacement figures and update the figure caption with source information. If applicable, please specify in the figure caption text when a figure is similar but not identical to the original image and is therefore for illustrative purposes only.

The following resources for replacing copyrighted map figures may be helpful:

USGS National Map Viewer (public domain): http://viewer.nationalmap.gov/viewer/

The Gateway to Astronaut Photography of Earth (public domain): http://eol.jsc.nasa.gov/sseop/clickmap/

Maps at the CIA (public domain): https://www.cia.gov/library/publications/the-world-factbook/index.html and https://www.cia.gov/library/publications/cia-maps-publications/index.html

NASA Earth Observatory (public domain): http://earthobservatory.nasa.gov/

Landsat: http://landsat.visibleearth.nasa.gov/

USGS EROS (Earth Resources Observatory and Science (EROS) Center) (public domain): http://eros.usgs.gov/#

Natural Earth (public domain): http://www.naturalearthdata.com/

6. Please upload a new copy of Figure 8 as the detail is not clear. Please follow the link for more information:  https://journals.plos.org/plosone/s/figures

7.  Please remove your figures from within your manuscript file, leaving only the individual TIFF/EPS image files, uploaded separately. These will be automatically included in the reviewers’ PDF.

8. If the reviewer comments include a recommendation to cite specific previously published works, please review and evaluate these publications to determine whether they are relevant and should be cited. There is no requirement to cite these works unless the editor has indicated otherwise.

[Note: HTML markup is below. Please do not edit.]

Reviewers' comments:

Reviewer's Responses to Questions

**Comments to the Author**

1. Is the manuscript technically sound, and do the data support the conclusions?

Reviewer #1: Yes

Reviewer #2: Partly

Reviewer #3: Partly

**********

2. Has the statistical analysis been performed appropriately and rigorously?

Reviewer #1: Yes

Reviewer #2: Yes

Reviewer #3: No

**********

3. Have the authors made all data underlying the findings in their manuscript fully available?

The PLOS Data policy

Reviewer #1: Yes

Reviewer #2: Yes

Reviewer #3: Yes

**********

4. Is the manuscript presented in an intelligible fashion and written in standard English?

Reviewer #1: Yes

Reviewer #2: Yes

Reviewer #3: Yes

**********

Reviewer #1: First of all, I would like to congratulate the authors for conducting this all important study on the burden of malignant melanoma.

This manuscript is a research article titled "Burden of malignant melanoma among the elderly population in East Asia from 1990 to 2023 and machine learning projection to 2040," which analyzes the trends and future projections of malignant melanoma (MM) burden among the elderly population in East Asia. This research highlights the urgent need for targeted public health interventions to mitigate the growing burden of malignant melanoma in East Asia's ageing population.

The objectives are well-defined and meet the SMART criteria, making them effective for addressing the research problem and providing actionable insights for public health strategies in East Asia.

The methodology is robust, scientifically sound, and appropriate for the research objectives. It uses reliable data sources, well-defined variables, and established statistical methods to analyse trends and project future MM burden. The study also acknowledges limitations, which is an essential aspect of scientific rigour.

The study has several strengths, including its use of a robust dataset, advanced statistical methods, and focus on a critical demographic group. However, it could be improved by addressing data limitations, exploring environmental and genetic factors in greater depth, and providing more detailed analyses of intervention effectiveness and cost-efficiency. Despite these weaknesses, the study provides valuable insights into the growing burden of malignant melanoma among the elderly in East Asia and offers actionable recommendations for public health strategies.

While the study is methodologically sound, potential biases related to data availability, selection, projections, and socioeconomic disparities should be considered when interpreting the findings. These biases do not invalidate the study but highlight areas where further research and data collection could improve the robustness and generalizability of the conclusions.

The study is well-suited for publication in PLOS ONE, provided the authors address the potential biases and limitations, ensure transparency, and strengthen their recommendations. Its focus on a significant public health issue and use of robust methodologies make it a valuable contribution to the field.

To ensure the study meets PLOS ONE’s standards, the authors should:

1. Discuss potential biases in data collection, reporting, and projections, and their implications for the study’s findings.

2. Provide a detailed analysis of the study’s limitations, including the reliance on secondary data and the exclusion of younger populations.

3. Offer more detailed and actionable public health strategies, including cost-effectiveness analyses and intervention evaluations.

4. Confirm that all data and methods are fully accessible and reproducible, as required by PLOS ONE’s data availability policy.

Reviewer #2: Summary:

This study analyzes the burden of Malignant Melanoma (MM) among older adults in East Asia from 1990 to 2023. The authors utilized data from the Global Burden of Disease Study 2023 and utilized joinpoint regression and time-series modeling to assess incidence, prevalence, mortality, and DALYs. The findings indicate substantial growth in the burden of MM, driven by population aging, with persistent sex-based and geographic disparities. While the study addresses a relevant public health issue, the current manuscript lacks technical rigor, containing significant discrepancies between reported data and presented figures, missing methodological details, and lacking a limitations section.

Major Comments

* General: The study mentions "Taiwan Province of China" alongside other Asian country names multiple times. I suggest either merging the China and Taiwan Province of China data and reporting it as China only, reporting it as "Taiwan" only, or deleting the "Taiwan Province of China" data from the manuscript entirely.

* Line 98: Reference 10 needs to be updated with an appropriate and reliable source.

* Line 104-105: The study states, “We employed the following ICD-10 diagnostic codes for case identification: C43–C43.9 and Z85.82–Z85.828.” Clarification is needed on whether the study also collected primary data to establish this case identification.

* Line 108: The authors note, “The study was exempted from ethical review because it relied exclusively on anonymized, open-access data.” Please clarify if the study formally sought IRB exemption from a review board and received an official exemption; if so, provide the official IRB exemption number.

* Line 197-220: The text uses highest, moderate, and lowest levels to categorize ASIR, ASPR, ASMR, and ASDR. The specific methods and thresholds for this classification must be added to the Methods section.

* Line 226-240: The drafted text contains several inaccuracies and misinterpretations of the provided Figures 2 and 3.

* Line 226-240: The text claims, "Mortality increased sharply after age 60, with male deaths peaking in the 55–59 age group." It is logically contradictory for mortality to increase sharply after age 60 while simultaneously peaking before 60 (at 55–59).

* Line 228: The manuscript states that incidence reached its maximum in the 75–79 age group. This assertion is incorrect based on the data displayed in both figures.

* Line 230: The text states prevalence peaks around ages 75–79 for both sexes. This is also incorrect in both figures.

* Line 250-251: The authors state "Male incidence rates... consistently exceeded female rates," but in Figure 4 A & B, female rates are consistently higher than male rates. The text also mentions a "significant widening beyond age 60," but the x-axis represents the year (1990–2023), not age.

* Line 255-256: The text attributes the rising prevalence to improved survival and the aging of the population. However, the line graph represents the Age-Standardized Prevalence Rate (ASPR), meaning any increase in the rate is inherently independent of population aging.

* Line 258: The study claims that ASMR (mortality rate) rose more significantly. While the absolute number of deaths increased significantly, the age-standardized mortality rate and DALYs in Figure 4 C & D appear relatively flat or show only a slight upward trend over the 30-year period.

* Line 274-277: The text claims incidence peaks in the "85–89 age group before slightly declining in those aged 90 years and older." Though there is a dip at the 90–94 age group, the visual data shows it rises sharply again in the 95+ year demographic.

* Line 285: The text states that "China bore the largest number of deaths in absolute terms." However, Figure 5 displays rates per 100,000, not absolute numbers.

* Line 287-288: The text mentions the "East Asian regional average displayed a consistent upward gradient." In Figure 4, the uppermost line represents the Eastern Asia regional average. The authors should explain how a regional average rate can mathematically be higher than the rates of all its individual constituent countries.

* Line 309-310: The text notes, "The ASDR followed a similar pattern, showing an overall upward trend." However, the corresponding data points in Figure 6 C & D display high volatility and periods of clear decline.

* Line 311: The manuscript claims DALYs peaked in growth between 2010 and 2018 (APC = 1.22%). There is no segment corresponding to 2010-2018; additionally, the 2012-2019 APC is -1.15, while the 1990-2000 APC is 1.27.

* Line 312-313: The text states, "Across all metrics, the burden was consistently higher in men than in women." In Figure 6 A & B, the visual data shows the exact opposite of this claim.

* Line 334-335: States that "mortality and DALY period effects declined after 2005, suggesting better prevention". But, in figures 7G and 7H, mortality and DALYs start declining from 1990.

* Line 360-361: States that "a gradual decline is anticipated for both mortality and DALY rates". But in 8E3 (Mortality) and 8E4 (DALYs) show a continuous upward trend that is projected through 2040. Also, states that incidence and prevalence are forecast to experience a moderate increase, and mortality/DALYs a gradual decline but I suggest make more specific to each graph.

* There is no limitations section included in the manuscript. Because this study is based entirely on secondary data derived from modeling, this must be transparently acknowledged and discussed as a major limitation.

Minor Comments

* Line 96: The phrase “longitudinal trends” can be replaced with “time trends” for better accuracy.

* Line 142-143: Please use the full name at the first use of the abbreviations APC and AAPC.

* Line 181-189: Move this entire section of text to position it after Table 1 and before Table 2.

* Line 224: Rename this sub-header to more accurately reflect the specific burden of MM being discussed.

* Line 238: The text states, "Overall, men consistently bore a heavier burden across all indicators." Please specify whether this refers to absolute numbers or standard rates, and clearly indicate which figures support this statement.

* Figure 1: Please remove the world map and use only an East Asia Map and/or country-specific maps of the included regions to ensure visual clarity.

* Figures 2 & 3: Figure 2 and Figure 3 currently have the exact same titles: "Age and sex differences in MM across East Asian countries in 2023.(A) Incidence rates.(B) Prevalence rates.(C) Death rates.(D) DALY rates". Title need to be specific to figures.

* Figure 7: label each figure to its corresponding measures.

* Figure 8: Is very blur and small, I could not read it.

Conclusion & Recommendation:

The manuscript addresses the relevant topic of malignant melanoma in East Asia but suffers from major structural issues, primarily contradictions between the text and the presented figures. The lack of a limitations section. Therefore, I think this paper should check its data analysis, figure and results sections and address all inconsistency. I think methods for this paper is very good but writing is substandard, it does not qualify PLOS One journal standard in current version. There need a major revision.

Reviewer #3: This is such a comprehensive analysis and breakdown of a rich data set. I agree with the general approach and think it could be replicated across different disease to generate insights about their management gaps and needs.

I am not a biostatistician, but I am concerned with the model y = ß0 + ß1.age + ß2.period + ß3.cohort. The reason is the collinearity between age, period and cohort. From experience with GBD, I’m not sure if it reports cohort. So, I’m assuming that cohort will be calculated as: period (in year) – age. Therefore, the 3 are interrelated. When fitting a model with all 3 as covariates, this creates a non-identifiability problem, i.e., multiple combinations could lead to the same y outcome. I am familiar with some apc models, e.g., the “epi” package in R, and would suggest those as alternatives…

Other minor comments:

• Overall, there seem to be a lot of discussion points that are intertwined with results (e.g., "This spatial variation suggests that regions with higher socioeconomic development and better healthcare infrastructure report greater disease detection and longer survival, thereby contributing to higher recorded incidence and prevalence rates." I suggest moving all those to the discussion section.

• The authors used 3 acronyms: EPAC, AAPC, & APC but those were not explicitly explained/distinguished. For example: In line 142: “APC” was used but was not explained, I’m assuming from the context that it is “annual percent change” and not “age-period-cohort”. Also, in line 143: AAPC vs line 131: EAPC, I’m assuming they mean the same thing: annual/estimated APC…

• I was under the impression that APC was reporting changes on the multiplicative scale, but only realized it was on the additive scale when the null value was reported to be 0 and noticed that some of the reported 95% CIs included negative values. With that, I’m not sure how to interpret the results: e.g., table 1, Eastern Asia, ASMR in 1990 = 2.05 and in 2023 = 2.17, EPAC = 50.70. I’m assuming that this value (50.70) is a percent, but it’s not clear to me what it means.

• Since many results are discussed in terms of gender disparity, it would make sense to further breakdown the location values by gender, and possibly age, just to track the actual breakdown in numbers.

• Fig 1 is hard to read, maybe resale to target locations only.

• Fig 2, the x axis labels are not clear, e.g., “The numbers of incidence” does that mean the number of prevalent cases?

• Fig 4, the bar plot is not a good visualization choice for numeric variables, I would replace with possibly dotted or dashed lines.

• In multiple contexts, it wasn’t clear for me if the word “burden” was meant to target the specific metric or all 4 indicators? If the latter, maybe switch to something like "epi indicators" maybe? For example, the Regional “Burden” Analysis section—in includes ASIR and ASPR analyses as well…

• Line 114 "All burden indicators were age-standardized against the GBD 2023 global standard population and reported as per 100,000 rates via direct standardization to account for varying population structures", is this describing a process done by the authors or GBD data?

• When age standardizing, one cannot attribute effects to aging, yet a lot of inferences were attributed to aging and its effects.

**********

what does this mean?). If published, this will include your full peer review and any attached files.

If you choose “no”, your identity will remain anonymous but your review may still be made public.

**Do you want your identity to be public for this peer review?** For information about this choice, including consent withdrawal, please see our Privacy Policy

Reviewer #1: **Yes:**  U S H GAMAGE

Reviewer #2: No

Reviewer #3: No

**********

[NOTE: If reviewer comments were submitted as an attachment file, they will be attached to this email and accessible via the submission site. Please log into your account, locate the manuscript record, and check for the action link "View Attachments". If this link does not appear, there are no attachment files.]

To ensure your figures meet our technical requirements, please review our figure guidelines: https://journals.plos.org/plosone/s/figures

You may also use PLOS’s free figure tool, NAAS, to help you prepare publication quality figures: https://journals.plos.org/plosone/s/figures#loc-tools-for-figure-preparation.

NAAS will assess whether your figures meet our technical requirements by comparing each figure against our figure specifications.

# Author comment: Author response to Decision Letter 1

**DOI:** 10.1371/journal.pone.0357767.r002

11 Aug 2026

yes

Attachment Submitted filename: Response to Reviewers 3.docx

# Peer review: Decision Letter 1

**DOI:** 10.1371/journal.pone.0357767.r003

**Authors:** Pengpeng Ye

23 Aug 2026

Burden of malignant melanoma among the elderly population in East Asia from 1990 to 2023 and machine learning projection to 2040

PONE-D-25-66779R1

Dear Dr. Wang,

We’re pleased to inform you that your manuscript has been judged scientifically suitable for publication and will be formally accepted for publication once it meets all outstanding technical requirements.

Within one week, you’ll receive an e-mail detailing the required amendments. When these have been addressed, you’ll receive a formal acceptance letter and your manuscript will be scheduled for publication.

An invoice will be generated when your article is formally accepted. Please note, if your institution has a publishing partnership with PLOS and your article meets the relevant criteria, all or part of your publication costs will be covered. Please make sure your user information is up-to-date by logging into Editorial Manager at Editorial Manager® and clicking the ‘Update My Information' link at the top of the page. For questions related to billing, please contact billing support.

If your institution or institutions have a press office, please notify them about your upcoming paper to help maximize its impact. If they’ll be preparing press materials, please inform our press team as soon as possible -- no later than 48 hours after receiving the formal acceptance. Your manuscript will remain under strict press embargo until 2 pm Eastern Time on the date of publication. For more information, please contact onepress@plos.org.

Kind regards,

Pengpeng Ye

Academic Editor

PLOS One

Additional Editor Comments (optional):

Reviewers' comments:

Reviewer's Responses to Questions

**Comments to the Author**

Reviewer #1: All comments have been addressed

Reviewer #2: All comments have been addressed

**********

2. Is the manuscript technically sound, and do the data support the conclusions?

Reviewer #1: Yes

Reviewer #2: (No Response)

**********

3. Has the statistical analysis been performed appropriately and rigorously?

Reviewer #1: Yes

Reviewer #2: (No Response)

**********

4. Have the authors made all data underlying the findings in their manuscript fully available?

The PLOS Data policy

Reviewer #1: Yes

Reviewer #2: (No Response)

**********

5. Is the manuscript presented in an intelligible fashion and written in standard English?

Reviewer #1: Yes

Reviewer #2: (No Response)

**********

Reviewer #1: The authors have addressed the reviewer comments thoroughly and effectively. Their responses are detailed, transparent, and directly linked to manuscript revisions. They have made substantial improvements in methodological clarity, data presentation, and the articulation of limitations and public health implications. The manuscript now better aligns with the reviewers' expectations for rigor, clarity, and reproducibility.

Reviewer #2: (No Response)

**********

what does this mean?). If published, this will include your full peer review and any attached files.

If you choose “no”, your identity will remain anonymous but your review may still be made public.

**Do you want your identity to be public for this peer review?** For information about this choice, including consent withdrawal, please see our Privacy Policy

Reviewer #1: **Yes:**  UDAYA SAMANTHILAKA HATTOTUWA GAMAGE

Reviewer #2: No

**********

# Editor report: Acceptance letter

**DOI:** 10.1371/journal.pone.0357767.r004

**Authors:** Pengpeng Ye

PONE-D-25-66779R1

PLOS One

Dear Dr. Wang,

I'm pleased to inform you that your manuscript has been deemed suitable for publication in PLOS One. Congratulations! Your manuscript is now being handed over to our production team.

At this stage, our production department will prepare your paper for publication. This includes ensuring the following:

* All references, tables, and figures are properly cited

* All relevant supporting information is included in the manuscript submission,

* There are no issues that prevent the paper from being properly typeset

You will receive further instructions from the production team, including instructions on how to review your proof when it is ready. Please keep in mind that we are working through a large volume of accepted articles, so please give us a few days to review your paper and let you know the next and final steps.

Lastly, if your institution or institutions have a press office, please let them know about your upcoming paper now to help maximize its impact. If they'll be preparing press materials, please inform our press team within the next 48 hours. Your manuscript will remain under strict press embargo until 2 pm Eastern Time on the date of publication. For more information, please contact onepress@plos.org.

You will receive an invoice from PLOS for your publication fee after your manuscript has reached the completed accept phase. If you receive an email requesting payment before acceptance or for any other service, this may be a phishing scheme. Learn how to identify phishing emails and protect your accounts at https://explore.plos.org/phishing.

If we can help with anything else, please email us at customercare@plos.org.

Thank you for submitting your work to PLOS One and supporting open access.

Kind regards,

PLOS One Editorial Office Staff

on behalf of

Dr. Pengpeng Ye

Academic Editor

PLOS One
